# A Computational Framework for Analyzing Stochasticity in Gene Expression

**DOI:** 10.1371/journal.pcbi.1003596

**Published:** 2014-05-08

**Authors:** Marc S. Sherman, Barak A. Cohen

**Affiliations:** 1 Computational and Molecular Biophysics, Washington University in St. Louis, St. Louis, Missouri, United States of America; 2 Center for Genome Sciences, Department of Genetics, Washington University in St. Louis, St. Louis, Missouri, United States of America; Rutgers University, United States of America

## Abstract

Stochastic fluctuations in gene expression give rise to distributions of protein levels across cell populations. Despite a mounting number of theoretical models explaining stochasticity in protein expression, we lack a robust, efficient, assumption-free approach for inferring the molecular mechanisms that underlie the shape of protein distributions. Here we propose a method for inferring sets of biochemical rate constants that govern chromatin modification, transcription, translation, and RNA and protein degradation from stochasticity in protein expression. We asked whether the rates of these underlying processes can be estimated accurately from protein expression distributions, in the absence of any limiting assumptions. To do this, we (1) derived analytical solutions for the first four moments of the protein distribution, (2) found that these four moments completely capture the shape of protein distributions, and (3) developed an efficient algorithm for inferring gene expression rate constants from the moments of protein distributions. Using this algorithm we find that most protein distributions are consistent with a large number of different biochemical rate constant sets. Despite this degeneracy, the solution space of rate constants almost always informs on underlying mechanism. For example, we distinguish between regimes where transcriptional bursting occurs from regimes reflecting constitutive transcript production. Our method agrees with the current standard approach, and in the restrictive regime where the standard method operates, also identifies rate constants not previously obtainable. Even without making any assumptions we obtain estimates of individual biochemical rate constants, or meaningful ratios of rate constants, in 91% of tested cases. In some cases our method identified all of the underlying rate constants. The framework developed here will be a powerful tool for deducing the contributions of particular molecular mechanisms to specific patterns of gene expression.

## Author Summary

Proteins, the molecular machines encoded by our genes, serve essential roles in every living cell. Investigators were therefore surprised to find widely variable levels of a particular protein within populations of genetically identical cells. This variation in protein level, called stochasticity, arises from the chemical nature of the processes that underlie protein production. The “central dogma” of biology dictates that the DNA encoding a particular gene transmits information via RNA to molecular factories called ribosomes in order to create proteins. Each step in transcription and translation introduces some variation, or stochasticity, into the production of the protein. In the current work, we tackled how one might learn more about the machinery responsible for creating proteins by the character of the stochasticity in the central dogma process. We find that many different mechanisms can explain any given stochastic protein signature. Even though there were many explanations for any particular pattern of stochasticity, the set of explanations still inform on how a given gene creates its protein. Our mathematical and computational framework will permit others to better understand how the machinery that expresses genes works. This, in turn, will enable investigators to better predict how a given mutation is likely to affect gene expression.

## Introduction

Stochasticity in transcription and translation produces fluctuations in both RNA [1]–[4], and protein [5]–[16]. On a population level these fluctuations manifest as a distribution of RNA and protein counts across cells. Both protein and RNA distributions are thought to contain information about the molecular processes governing transcription and translation, though how much information is unclear [17]. Learning the mechanistic details of a gene's expression from its stochastic signature requires (1) a method to measure cell-to-cell expression variability experimentally, (2) an explanatory stochastic model that simulates this variability *in silico*, and (3) a method for fitting the model parameters from experimental data.

**Figure 1:**
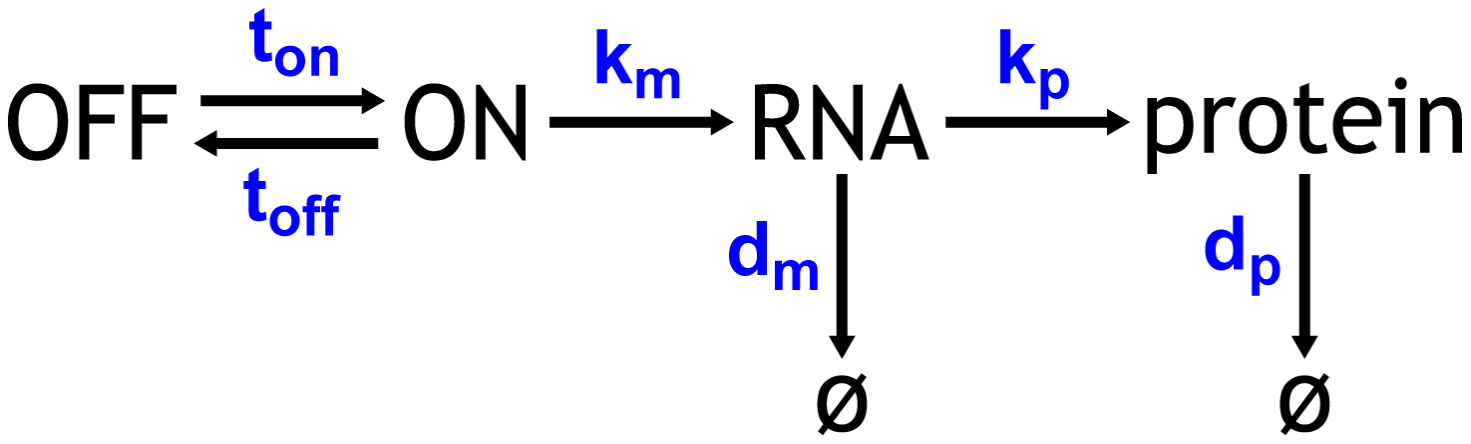
Central dogma model of gene expression with gene ON and OFF states.

There are consensus methods for accomplishing tasks (1) and (2). Investigators routinely measure protein expression stochasticity by recording reporter gene fluctuations with flow cytometry [5], [11], [14], [16], [18] or microscopy [1], [3], [8], [19]–[21]. The result is a protein count distribution that reflects cell-to-cell variation in gene expression. To simulate this cell-to-cell variation *in silico*, investigators developed a stochastic model of gene expression (Fig. 1), which has proven to be an effective abstraction of the central dogma [1], [3], [5], [8], [10], [14], [20], [22]. This model is parameterized by six central dogma rate constants (CDRCs) that govern a gene's ON (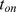) and OFF (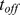) transitions, transcription from the active state (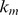), translation of RNAs (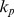), and degradation of RNA (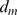) and protein (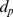). With a specific set of CDRCs the gene expression model depicted in Fig. 1 can be simulated with the Gillespie algorithm [23] to produce the corresponding protein count distribution [18], [24]–[29].

Task (3), fitting the model of stochastic gene expression to protein distributions, has no general solution. One possible approach would be to test candidate CDRC sets by Gillespie-simulating their corresponding distributions until a CDRC set that best approximates an experimentally measured distribution is identified. Several investigators have shown the utility of this approach, however, the systems being modeled comprise RNA expression only, where the number of molecules is low [4], [30]. The well-documented inefficiency of the Gillespie algorithm at even modestly high molecule counts [31]–[36] renders this approach untenable for parameter estimation of protein distributions, where the average protein expression of single genes exceeds 12000 proteins per cell [37].

An alternative approach is to analytically solve for the shape of the protein distribution as a function of the CDRCs. This approach has yielded some elegant solutions; however, (1) the analytical solutions generally involve hypergeometric or gamma functions, themselves ill-suited for parameter estimation, and more problematically (2) each solution makes specific assumptions about a gene's expression [17], [22], [25], [28], [38]–[40]. Some methods are valid only when protein count is high [25], [39], while most require RNA degradation to be orders of magnitude faster than protein degradation [22], [25], [28], [38], [40]. Several only apply when genes do not have an OFF state [9], [25] or the ON-OFF transitions are rapid [39]. Three approaches do not model RNA fluctuations [17], [38], [40]. The result is a fundamental limitation in the applicability of these methods, since it is usually unknown beforehand whether a particular gene conforms to the basic assumptions of these methods.

Even an assumption-free analytical solution to the protein distribution may not adequately solve the fitting problem. Munsky *et al* determined that degeneracy in the solution space of CDRCs means that ratios of the CDRCs, but not the CDRCs themselves, can be extracted from steady state protein distributions, and further recommends temporal measurements for pinpointing individual CDRC values [41]. Similarly, Ingram, Stumpf and Stark demonstrated that many different combinations of CDRCs can give rise to the same translational burst distribution [33]. The authors suggested supplementing the burst distribution with the steady state protein distribution, but were hindered in part by the inefficiency of the Gillespie algorithm [33]. Any method for determining the CDRCs that underlie protein distributions must account for the expected degeneracy of CDRCs that can produce a given protein distribution. Given this degeneracy, it is an open question how much information any given protein distribution contains about the CDRCs that underlie its shape. Mechanistic information about the processes that produce an observed protein distribution will most likely come from analyzing ensembles of solutions that fit a particular protein distribution.

In this work we address how much information is contained in protein distributions. The principal result is an assumption-free solution to the steady state protein distribution. Our approach consists of two parts: (1) analytical solutions to the first four moments of the protein distribution, and (2) an efficient, exhaustive fitting algorithm that returns ensembles of CDRC sets that map to a particular set of moments. The main power of our approach is that it returns all CDRC solution sets that are consistent with a given protein distribution. These solution set ensembles were always informative. Even in cases where we observe degeneracy in both the individual CDRCs and their ratios, the set of solutions provides mechanistic information about gene behavior, for example distinguishing between genes undergoing transcription bursts from those that transcribe constitutively.

We compared our method directly to the Friedman analytical solution [25]. While the model solved analytically by Friedman *et al* does not incorporate an OFF state like other solutions [22], [28], [29], only the Friedman result enables rapid estimation of the causative CDRC ratios. Investigators recently took advantage of this result to infer 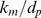 and 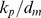 from protein distributions measured by flow cytometry [42]–[44]. We found that in the restrictive regime where the Friedman assumptions hold, our method not only identifies both Friedman ratios, but often obtains estimates for quantities inaccessible to the Friedman method, including the average RNA count, 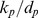, and in some cases even individual CDRCs.

Without assuming any regime, we find that supplementing our fitting algorithm with experimentally determined limits for each CDRC permits some CDRCs to be obtained individually. More often, CDRC ratios are well conserved among the ensemble of solution outcomes. We can identify at least one CDRC in 40% of regimes tested, and at least one CDRC ratio in 91%. Our methodology provides a general, assumption-free framework for extracting information from protein distributions. We anticipate that our approach will be a powerful tool for quantitatively characterizing the molecular machinery that underlies gene expression.

## Results

### Computing moments from Central Dogma Rate Constants

The stochastic behavior of the central dogma model (Fig. 1) is exactly described by the chemical master equation (CME), Eq. 1 & 2.
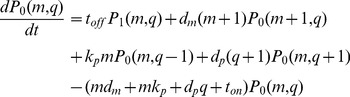
(1)

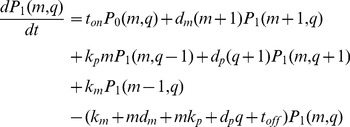
(2)Here we separated the CME into two equations that describe the system in its ON (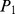) and OFF (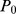) states. 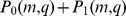 is the probability that a single cell will contain 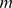 RNAs and 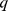 proteins. Thus, the probability space is a joint distribution for all possible combinations of 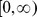 RNAs and 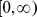 proteins for each promoter state.

The challenge is to use the CME to determine the CDRCs that underlie an experimentally determined protein distribution. With the CME model and a set of input CDRC parameters, a protein distribution is determined by either Gillespie-simulating the CME, or by solving the CME analytically. However, as discussed above, numerical simulations are prohibitively expensive, and the analytical solutions are valid only under restrictive assumptions.

To address this challenge we chose a hybrid approach that takes advantage of the moments of a protein distribution as descriptors of the protein distribution's shape. A significant body of work establishes the relationship between the CDRCs and the first two moments, mean and variance [5], [9], [10], [13], [15], [25], [26], [28], [39], [45]. However the mean and variance alone do not sufficiently characterize the shape of an experimentally measured protein distribution [24], [41], [46]. We therefore extended previous work by solving for the third (skewness) and fourth (kurtosis) steady state, central moments of protein distributions as functions of the six CDRCs.

To analytically derive protein skewness and kurtosis, we adopted the approach of Sánchez and Kondev in which the 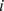-th moment is computed by multiplying both Eq. 1 and 2 by 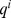 and summing over all possible 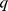 [45]. Instead of assuming instantaneous geometric distributions of protein, however, we explicitly modeled protein translation. When translation is treated in this way, the 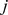-th protein moment 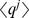 depends on all RNA-protein covariate moments whose sum equals 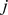. The result is the following expansion for the 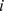-th and 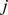-th moments and covariant moments between the RNA and protein distributions.
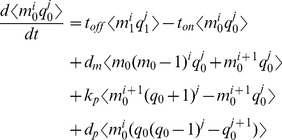
(3)

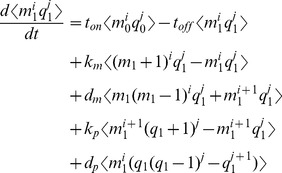
(4)


The result is a set of 28 linear ODE partial moment equations whose time derivatives we set to zero and solved simultaneously to obtain protein skewness and kurtosis. A complete derivation is provided in the Supplement.

The equilibrium equations for mean (Eq. 5) and variance (Eq. S66) agree exactly with previously published analytical results [47]. Equations for skewness and kurtosis are provided as implementations in both MATLAB (2012a, The MathWorks, Natick, MA) and C++ (Data S2). We checked these equations against Gillespie-simulated protein distributions generated using diverse sets of CDRCs. We then asked whether the sample moments of these simulated protein distributions agreed with our analytical moments. In all cases we found that the Gillespie simulations converged on our analytical solutions. An example convergence is shown in Fig. S1. Thus, we can express the first four moments of a protein distribution as functions of the CDRCs.

### Finding CDRC sets from moments

The results so far are exact equations relating the CDRCs to the moments of protein distributions. To determine the CDRCs that underlie an experimentally measured protein distribution we must solve the inverse problem; we must implement a fitting method that takes the moments of a protein distribution as input, and returns the best estimate of the causative CDRCs. When considering estimation procedures, we took under consideration a previous result that steady state protein distributions alone cannot contain enough information to directly fit CDRCs [41]. Corroborating that observation, Ingram *et al* found that even constraining on both translational burst size and one of the degradation rates, 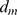 or 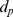, revealed degeneracy in the remaining unknown CDRCs [33]. Since standard fitting approaches behave erratically when solution spaces comprise flat or valley shaped minima, we were motivated to consider alternatives more robust to parameter space degeneracy.

The approach we took is to exhaustively test all of six-dimensional CDRC space within the physiological ranges for each CDRC. Physiological ranges for each CDRC were drawn from various genome-wide analyses of *S. cerevisiae*. The degradation and synthesis rates' ranges were set to be 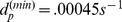, 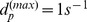, 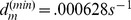, 
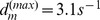
, 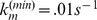, 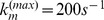, 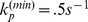, and 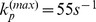 [11], [48]. From the few *in vivo* measurements of 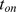 and 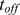 [8], [49] we extrapolated several orders of magnitude, setting these ranges to be 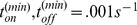, and 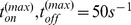.

Our brute force fitting routine gains efficiency by leveraging the fact that the expression for the mean is simple (Eq. 5). Given physiological ranges for each CDRC and an equation for the mean, each choice for the value of one CDRC analytically limits the values of yet-to-be-assigned CDRCs. When the algorithm reaches the sixth and last CDRC, only one value of this CDRC satisfies the mean equation. In this way we reduce the complexity of our CDRC search by at least one dimension. The exact algorithmic and mathematical details of this Analytically Constrained Exhaustive Search (ACES) routine are a central result of this work, and are presented in Materials and Methods and continued in the Supplement. We also provide an ACES implementation in C++ (Data S2).

The ACES algorithm is efficient. If we examine 83 guesses across the range for each CDRC, the ACES algorithm tests 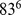, or 327 billion possible parameter sets, and returns all solutions that satisfy the input moments within some error tolerance. Here we chose a cutoff of <1%, though we envision adjusting the tolerance to be consistent with measured experimental error in the moments. A typical ACES execution takes approximately one minute at a resolution of 83. Testing such a large number of CDRC sets is only possible because (1) calculating the moment objective functions is fast, (2) we check the most efficiently calculated objective function first (variance), and the least efficient objective function (kurtosis) last, thus our most expensive function is called least frequently, and (3) we only test candidate CDRC sets that we already know satisfy the mean moment equation exactly. It is (3) that contributes most substantially to the efficiency of ACES.

### Generating a library testing all parameter regimes

With a set of equations in hand that relate the CDRCs to the moments of protein distributions, and an algorithm that uses those equations to fit CDRCs to sample moments, we now have a method for determining the CDRCs that underlie an experimentally measured protein distribution. To evaluate the utility of our method, we tested its ability to recover CDRC values from protein distributions defined by known CDRC sets.

We chose CDRC sets to test keeping in mind that a key drawback with other methods is that they only apply in specific parameter regimes. Our method makes no such assumptions, and so should be applicable in every regime of CDRC values. To test “every regime” in a course-grained manner, we constructed a representative library of CDRC sets. Because each of the six CDRCs can vary over several orders of magnitude, we selected five values spaced evenly in log space across the physiological range for each CDRC (Table S1). Our initial library contains all possible combinations of the 5 values for each of the 6 CDRCs, or 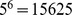 CDRC sets. We then removed all CDRC sets where the associated mean protein count was less than 17 or greater than 100,000, leaving 8053 CDRC sets. The lower limit was chosen as the lower limit of detection for fluorescent protein molecules, since our method is only applicable when fluorescence is measurable experimentally [50]. The upper limit was chosen arbitrarily to capture >98% of *Saccharomyces cerevisiae* genes [37].

**Figure 2:**
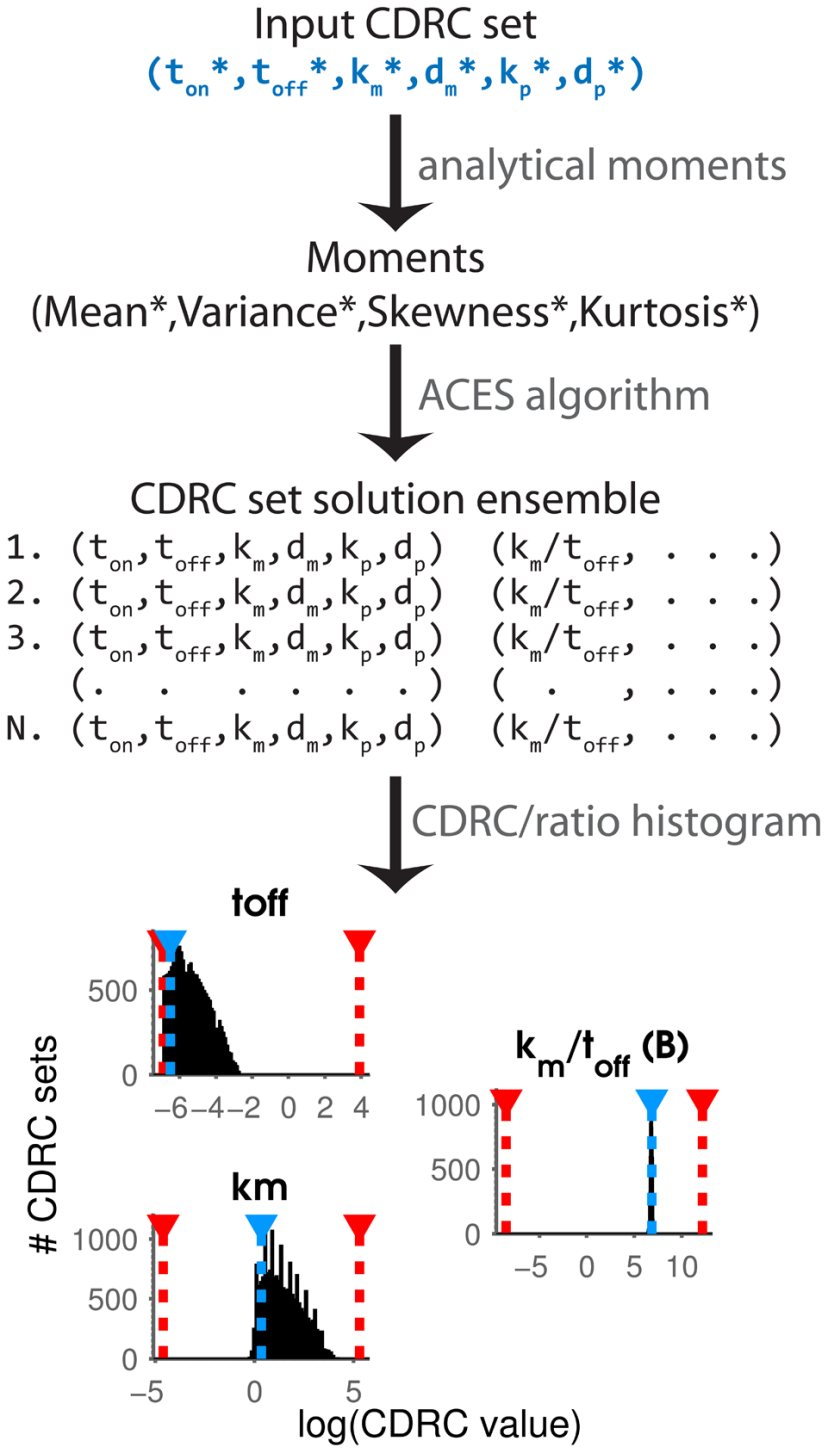
CDRC estimation pipeline. At the top, a library member input CDRC set (blue,*) is used to compute its corresponding moments (*). ACES takes as input only the moments, and returns all CDRC sets that correspond to those moments (the “ensemble”). To visualize this result, a histogram is generated for each CDRC or ratio column-wise. In the resulting histograms, physiological range of the parameter (red, dashed), the input value (blue dashed), and the ensemble solution values (black) are plotted for the tested parameter space (x-axis). On the bottom left, histograms for the CDRCs 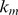 and 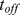 span half their physiological range, but their ratio is constrained to a single value (right, burst size).

To assess how well ACES recovers CDRCs across all parameter regimes, we applied ACES to each of the 8053 input CDRC sets. For each CDRC set in the library, we computed the moments of its protein distribution using our analytical solutions, and gave these moments to our ACES algorithm as input. ACES then returned a list of solutions, where each solution is a set of values for each of the six CDRCs (Fig. 2). Every solution returned by ACES produces moments that match the input moments with <1% relative error for each moment.

The number of solutions returned by the method depended on the number of subdivisions probed by the ACES algorithm. At a resolution of 83 (each CDRC tested at values ranging over 83 divisions across its physiological range), we find that 7555 of 8053 library member inputs result in at least one solution within the tolerated error level. Of the remaining 498, increasing the resolution to 127 resulted in solutions for all but 48 input sets, and took approximately 3 minutes per run at this resolution. The remaining 48 were extremely resistant to increases in resolution, however, simply reversing the order in which ACES tests parameters allowed us to find solutions for all of these remaining input sets at low resolution (See Supplement, section S3.2).

We find multiple candidate solutions for every input set. The number of solutions linked to any given CDRC set varied widely, with some producing dozens of solutions, and others generating tens of thousands. The median number of output solutions for a CDRC input set was 29286 solutions. This seems like a large number, but when put in the context that each ACES run tests hundreds of billions, or trillions of parameters sets, this number of solutions represents only a tiny fraction of tested CDRC sets. We considered two possible explanations for the multiplicity of solution sets obtained for every input set. (1) A previous analytical result suggests that CDRC ratios but not their individual values are recoverable from the steady state distribution [41]. Thus, every combination of CDRCs conserving a particular ratio will come out of our solution set. Although this explanation likely contributes to the degeneracy we observed, a competing explanation is that (2) four distribution moments inadequately describe the shape of protein distributions. We sought to distinguish between these two possibilities.

#### Maximally dissimilar CDRC sets with the same moments produce the same distribution

**Figure 3:**
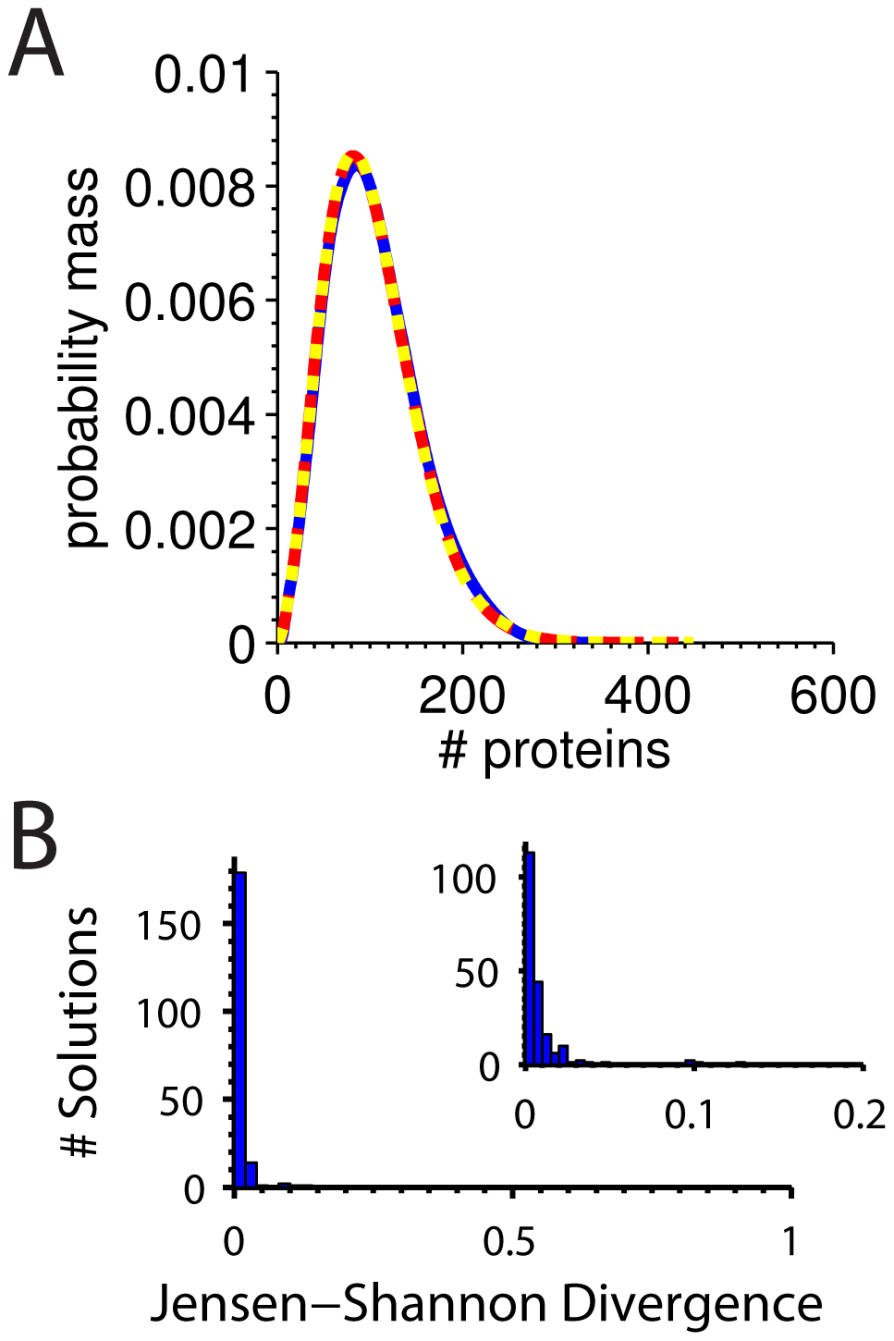
Distributions that correspond to CDRC sets with identical moments. **A** Two CDRC solution sets maximally different from one another in log-Euclidean space are plotted in (solid) blue (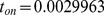, 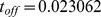, 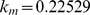, 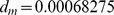, 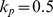, 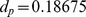) and (long-dashed) red (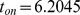, 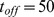, 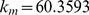, 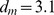, 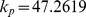, 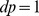). These candidate CDRC solution sets map from ACES fitting of library member 6328, plotted in (short-dashed) yellow from CDRC set (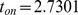, 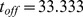, 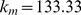, 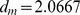, 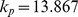, 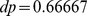) **B** Distribution of Jensen-Shannon divergences of maximally-distant CDRC candidate solution sets from their reference CDRC input set. (Inset) Zoom on the x-axis.

We asked how well moments approximated their corresponding protein count distribution by Gillespie-simulating examples of output CDRC sets, along with their input set, and comparing the resulting distributions. Given the abundance of output sets for every input set, we sought specific cases where the ensemble of output sets was least informative. We chose the 100 worst fit library members, where neither CDRCs nor ratios were well captured, and further identified CDRC output sets within each solution ensemble that were the farthest apart in log-Euclidean space. For example, the following two CDRC sets give rise to almost identical mean, variance, skewness, and kurtosis: (a) 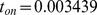, 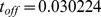, 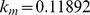, 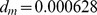, 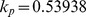, 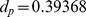, (b) 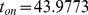, 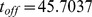, 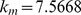, 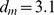, 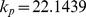, 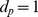. Almost every CDRC value between these two CDRC sets differs by several orders of magnitude. We then Gillespie-simulated both CDRC sets and their associated input set to generate complete protein distributions. We found that the protein distributions for all 100 “maximally distant pairs” matched each other and their input distributions (Figs. S2–S6). An example of a maximally distant CDRC pair and its reference distribution are plotted in Fig. 3A.

To quantify the differences between distributions, we computed the Jensen-Shannon divergence [51] between distributions, which is the symmetrized version of the Kullback-Leibler directed divergence used by Shahrezaei *et al* for similar purposes [22]. Jensen-Shannon divergence (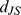) values have an intuitive interpretation; if 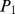 represents one probability distribution, and 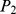 the second, 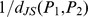 roughly estimates the number of samples one would have to draw to determine from which distribution (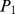 or 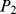) the values were taken [52]. Thus, Jensen-Shannon divergence ranges between 0, indicating that two distributions are identical, to 1, characterizing a pair of distributions as non-overlapping. We found that maximally different CDRC solution sets produce identical protein distributions (Fig. 3B). The median Jensen-Shannon divergence between protein distributions derived from maximally different CDRC solutions sets and the reference distribution is .0043, a number which is very close to the median divergence between Gillespie-simulated replicates of the reference distribution, .0033. This result indicates that independent replicates of one parameter set are essentially indistinguishable from distributions generated from maximally distant parameter sets that share the same moments.

We then asked whether the 5th sample moment could distinguish between distributions that shared identical first four moments (Supplement). We found that among the maximally distant sets, their fifth moments were indistinguishable. Taken together, these results confirm that the many solutions we obtain from ACES arise solely from CDRC degeneracy and not from the inadequacy of the first four moments to capture distribution shape.

#### Protein distribution shape informs on molecular mechanism

With a rigorous way of identifying the ensemble of CDRC sets that correspond to a particular protein distribution, we surveyed how well these ensembles inform on the molecular mechanisms that underlie the shape of protein distributions. For each CDRC output set, we computed ratios that represent physically relevant quantities, including: 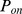 (probability that a promoter is active, 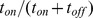), 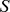 (RNA synthesis rate, 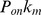), 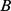 (burst size, number of RNAs produced per ON duration, 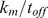), 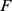 (burst frequency, frequency of promoter transitions between the ON and OFF states, 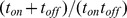), 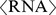 (average count of RNAs, 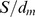), 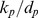 (ratio of average protein count to average RNA count), 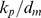 (translational burst size [25]), and 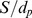 (number of RNAs produced per cell cycle when protein degradation is dominated by dilution and there are no ON-OFF transitions [25]).

**Figure 4:**
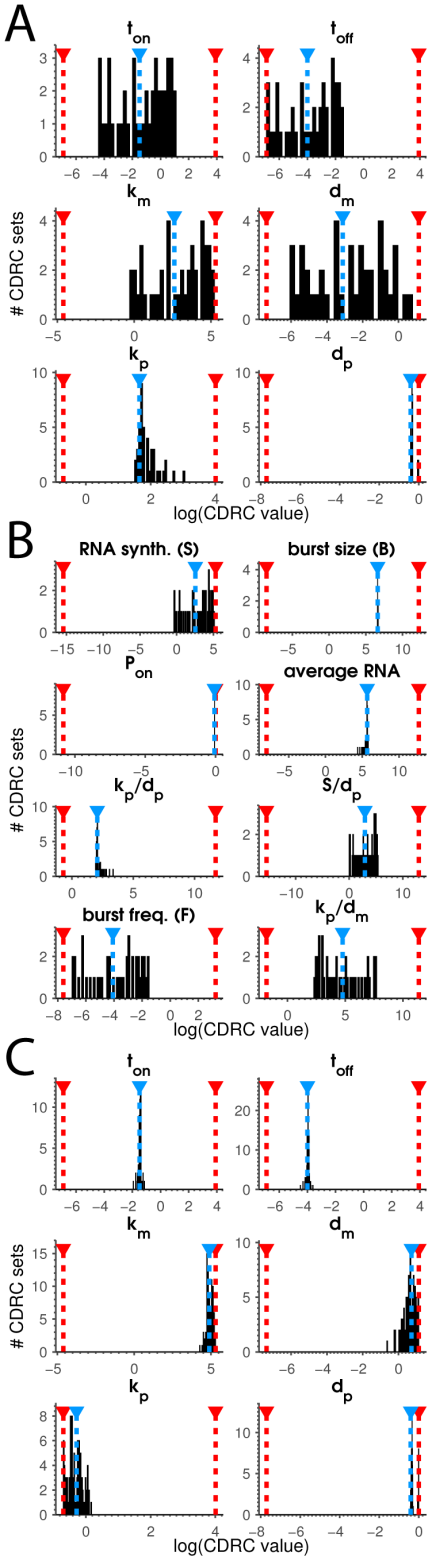
Fitting CDRCs from moments. Plotted is a histogram of CDRC solutions separated by parameter. Blue lines show the value of the input CDRC parameter, and red lines denote each CDRC's minimum and maximum value. **A** CDRC solution sets for fitting library member 3515 with CDRC input values (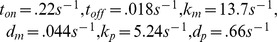), demonstrate only some parameters are well fit from four moments in this example. **B** Solution CDRC ratios from the same output set in (A) (library member 3515) reveal some CDRC ratios are well identified. **C** CDRC solution sets for fitting library member 3585, with input CDRC values (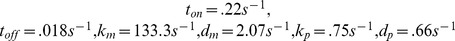), demonstrate all parameters are well fit from four moments in this example.

To develop our intuition, we plotted individual CDRC set output ensembles as histograms (Figs. 2, 4). Consistent with others' predictions [33], [41], we found that many different individual CDRC values map to the same distribution, while some CDRC ratios are exactly conserved in the solution ensemble. This is illustrated at the bottom of Fig. 2, where histograms for 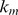 , 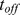 , and 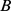 reveal the range and frequency of each CDRC value in the solution set. While 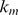 and 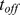 may assume many different values, their ratio (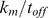) is constrained across every solution, indicating that the transcriptional burst size is a defining feature of this protein distribution. The solution ensemble for all CDRCs in a different example reveals that some values for each CDRC are not allowed by the distribution shape (Fig. 4). For example, 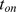 is restricted to the middle range of its possible values. We plotted histograms of the CDRC ratios in Fig. 4B from the same input set as in Fig. 4A, revealing that several ratios are exactly inferred from the distribution shape (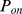 , 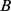, 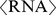). Contrary to our expectations, ACES also returned many examples of individual CDRCs themselves being well captured by the distribution shape. For example, Fig. 4A illustrates that 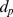 is well-estimated. In rare cases every CDRC solution clustered exactly on top of the associated input CDRC value (Fig. 4C). Equally rarely we discovered library members where neither CDRCs nor ratios were reasonably estimated (Fig. S7).

Examining each CDRC, or CDRC ratio ensemble as a histogram was the most informative way of viewing our results, however, this approach was not amenable to analyzing a library of 8053 members. To better grasp what ACES learned about CDRCs or ratios across the library, we developed a metric of fitness computed for each CDRC parameter. We tried many different metrics, and ultimately chose median log distance (MD) as our metric of fitness. To compute MD, we first calculated the log distances of each solution value for each CDRC compared to the known input value. That is, for the 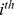 solution, 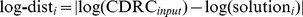. We then take the median of this list of distances as the MD value. Thus, lower MD values correspond to closer clustering of output values on the true solution. We emphasize that this metric is for survey purposes only–the value of ACES is in producing all possible CDRC solutions consistent with a distribution.

**Figure 5:**
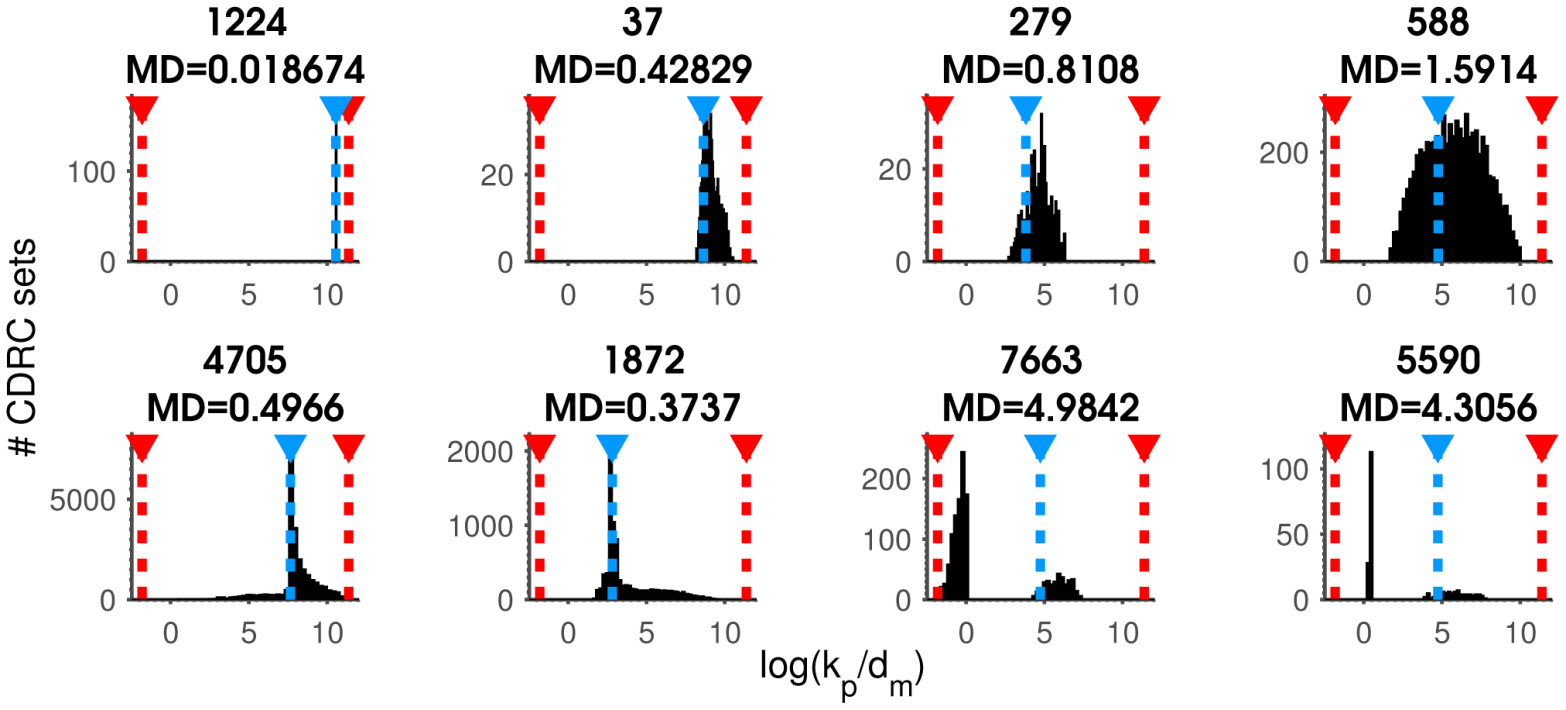
MD as a metric of fitness. Solution histograms for the CDRC ratio 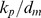. Titles show the library input set number above, and the corresponding MD for the histogram below. (Top row) Increasing MD values typically correspond to increasing distribution widths. (Bottom row, left 2 panels) Poorly estimated ratios with misleadingly low MD values. (Bottom row, right 2 panels) Informative CDRC ratio histograms with misleadingly high MD values.

With this purpose in mind, we arbitrarily defined a successful fit as an MD<.75, corresponding to the median solution for a particular CDRC or ratio being less than an order of magnitude away from the true (input) parameter value. We find that the MD metric generally aligns with the shape of a solution histogram; very small MD values indicate a spike of solutions immediately on top of the true solution, while increasing MD values correspond smoothly to increasingly wide distributions about the true solution (Fig. 5, top row). Distribution shapes not well captured by the MD metric include cases where the majority of solutions map to the true solution, while a minority of solutions appear elsewhere (Fig. 5, bottom row, left two panels), as well as cases where the CDRC or ratio may only take on two values, but the values are very different (Fig. 5, bottom row, right two panels).

With reasonable confidence in our survey measure, we computed the MD for every CDRC and ratio in each solution output set. The results of this analysis for every library input set are recorded in Data S1, while a summary of the results broken down by CDRC or ratio are presented in column MVSK, Table 1. Overall we can obtain at least one CDRC value or ratio in 91% of the library members. Although only 89 distributions allow inference of all six CDRCs, our analysis revealed 3276 of the CDRC input sets contain at least one correct CDRC fit. The breakdown in CDRCs identified was as follows: 13.1% for 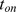, 7.1% for 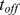, 6.8% for 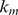, 16.2% for 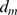, 21.1% for 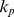, 18.2% for 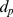. Among ratios, ACES identified 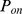 most frequently, even when the individual values of 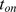 and 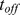 were poorly fit. 2396 input parameter sets correctly identify 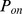 (MD = 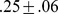) when both 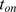 and 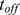 solution distributions were wide (MD>.75). On average, we fit .82 CDRCs and 2.55 ratios per library member, which we found to be remarkably informative given the overall degeneracy in protein distributions.

**Table 1:** Summary of CDRC fitting across input CDRC libraries.

	MVSK	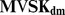	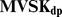	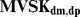	MV	MVS	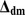	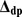	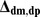	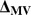	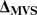
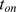	1.88	1.52	1.54	0.92	2.28	2.02	−0.36	−0.33	−0.96	0.40	0.14
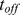	3.08	2.90	2.83	2.55	4.03	3.28	−0.19	−0.25	−0.53	0.94	0.20
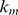	2.06	1.67	1.67	1.15	2.80	2.30	−0.40	−0.39	−0.92	0.74	0.24
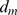	1.64	-	1.15	-	2.44	1.82	-	-0.49	-	0.80	0.18
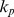	1.16	0.67	0.70	0.32	1.31	1.23	−0.49	−0.46	−0.84	0.15	0.07
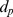	1.58	1.05	-	-	2.47	1.69	−0.53	-	-	0.90	0.11
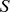	1.58	0.83	0.91	0.32	2.45	1.83	−0.75	−0.68	−1.26	0.86	0.24
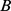	2.56	2.22	2.32	2.09	3.93	2.93	−0.33	−0.23	−0.47	1.38	0.38
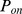	1.22	1.09	1.11	0.89	1.87	1.39	−0.13	−0.11	−0.33	0.65	0.17
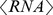	1.29	0.83	0.70	0.32	2.53	1.50	−0.46	−0.59	−0.97	1.24	0.21
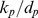	1.29	0.83	0.70	0.32	2.53	1.50	−0.46	−0.59	−0.97	1.24	0.21
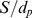	1.26	0.67	0.91	0.32	2.22	1.55	−0.59	−0.35	−0.94	0.96	0.29
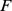	2.37	2.16	2.14	1.86	2.76	2.49	−0.22	−0.23	−0.51	0.39	0.11
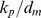	1.26	0.67	0.91	0.32	2.22	1.55	−0.59	−0.35	−0.94	0.96	0.29
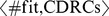	0.82/6	1.78/5	1.80/5	2.21/4	0.22/6	0.64/6	0.96	0.97	1.39	−0.60	−0.18
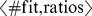	2.55	3.98	3.99	5.68	0.27	1.86	1.43	1.44	3.13	−2.28	−0.69

All columns correspond to average MD fit values across the same CDRC library members, fit under different conditions. MVSK corresponds to fitting on all four moments (mean, variance, skewness, and kurtosis), while MV and MVS correspond to fitting on mean-variance only, and mean-variance-skewness only, respectively. All values are reported in MD. Subscripted 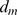 or 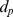 correspond to columns where those CDRCs were given to ACES *a priori*. 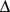 columns correspond to MD values in columns 2 through 6 minus the MD value for the reference column, MVSK, in column 1. 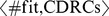 is the average number of CDRCs fit per input set according to our cutoff of MD<.75. In the relevant columns, CDRCs given as input are not included in the count of correct CDRCs. 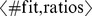 then corresponds to the number of CDRC ratios fit by the same critieria.

### CDRC inference improves when degradation rates are known

One way to reduce the complexity of CDRC-space is to experimentally measure some of the CDRCs that underlie a given protein distribution. In the context of this *in silico* study, measuring a CDRC corresponds to giving ACES one or multiple CDRCs at the outset. We analyzed the performance of ACES when given the values of the RNA and protein degradation rates, either separately or together. We chose the degradation rates because they are the easiest CDRCs to measure experimentally [11], [48], [53].

We re-ran our 8053 library of input sets giving ACES 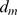, 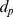, or both at the outset. As before, ACES returned solution sets for every input set, though ACES typically returned solutions on the order of a few seconds rather than 1–3 minutes at comparable resolutions.

Knowing 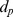 or 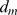 *a priori* improved ACES' fit of the remaining CDRCs and CDRC-ratios. When no CDRCs were known, .8/6 CDRCs and 2.55/8 ratios were fit per CDRC input set on average (column MSVK, Table 1). When 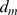 was given, 1.78/5 CDRCs and 3.98/8 ratios were fit on average (column MSVK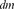). When 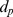 was given, these numbers are 1.80/5 CDRCs and 3.99/8 ratios (column MSVK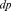). Constraining ACES with 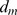 versus 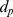 improved estimation of two to three other CDRCs or CDRC ratios. Both degradation rates indiscriminately improved estimation of the remaining unknowns, though estimation of 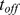 improved only modestly, while estimation of the synthesis rate (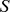) improved the most (columns 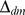, 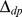, Table 1).

When ACES was given both degradation rate constants beforehand, we observed a dramatic improvement in CDRC and CDRC-ratio estimation (columns MSVK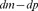, 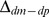, Table 1). The parameters 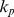, 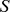, 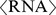 , 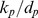, 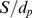, and 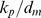 were essentially always measurable. On average 2.21/4 CDRCs and 5.68/8 ratios were fit in this library. Only 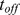 , and two ratios that depend on 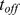 (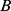 and 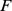) were not significantly improved by providing either or both degradation rate, an observation that will be explored further in a later section. Overall, our results suggest that experimentally determining 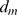 and 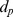 will greatly improve the estimation of the remaining CDRCs from experimentally measured protein distributions.

### Contribution of skewness and kurtosis to CDRC fitting

Higher moments contain diminishing information about the shape of a probability distribution. To determine if skewness and kurtosis contribute to ACES' fitting of the CDRCs, we performed two experiments on the exact same library of CDRC input sets as above. In the first, we modified ACES to fit only on the mean and the variance (column MV, Table 1), and in the second we fit on the first three moments (column MVS, Table 1).

We found that when ACES fits on mean and variance, on average only .22/6 CDRCs and .27/8 CDRC ratios were extracted per input set. When ACES was given the mean, variance, and skewness, it returned on average .64 CDRCs and 1.86 CDRC ratios for each input set. Thus, skewness contributed approximately .42 additional CDRCs and 1.59 CDRC ratios, while kurtosis added a modest but significant .18 CDRCs and .69 CDRC ratios per input. We conclude that skewness and kurtosis significantly contribute to the parameter fitting process, effectively quadrupling the number of CDRCs determinable per set, and increasing the number of CDRC ratios determinable by just under ten-fold.

### Distinguishing between bursting and non-bursting genes

Because a major challenge in the field is to identify the *cis*- and *trans*-acting machinery responsible for correlated transcriptional bursts, we asked whether our method distinguishes between CDRC sets that demonstrate bursting from sets that do not. First, since our library contains every possible CDRC regime, we attempted to identify which input sets exhibit transcriptional bursting. Second, we asked how well the burst parameter is detected by ACES, and whether certain regimes are more or less amenable to measuring the burst size from a protein distribution.

The Fano factor of the RNA distribution (
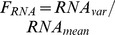
) distinguishes between constitutively active genes (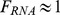) from bursting genes (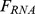
>1) [4], [26]. This is because in the limit of 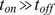, or if 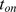 and 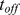 are both high, the RNA distribution's variance approaches its mean, and the Fano factor goes to 1 indicating constitutive RNA production. When we plot how well the burst parameter is fit versus 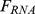, we find that near 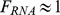 the average MD in all libraries is very high, but drops precipitously when 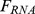 is slightly greater than 1 (Fig. S8A). Average MD of the burst parameter then slowly increases with increasing 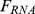. To gain some insight into this trend, we plotted how well the burst parameter was fit versus the value of the burst parameter itself (Fig. S8B). We observe that estimation of the burst parameter incrementally improves with increasing burst size until reaching an optimum corresponding to bursts of ones to tens of molecules. Above this, increasing burst size makes the burst parameter increasingly difficult to ascertain.

These data suggest that the high Fano factor regime corresponds to transcription of hundreds to thousands of transcripts per ON event, followed by long periods of gene quiescence. In this large burst regime, the burst parameter is difficult to infer precisely from the distribution. However, the ensemble of candidate solutions for 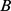 in these situations proves to be informative. Plotted in Fig. S9 are the forty highest Fano factor library members' burst parameter histograms. While some demonstrate exact inference of the burst parameter (305–307, 234–237), and others exhibit widely varying burst parameter values (637–645), all but one (638) of the forty reveal a burst size confined to 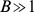, consistent with the behavior of the gene (Fig. S9). We conclude again that even when individual CDRCs or their ratios are not exactly inferred from the output of ACES, the shape of the solution ensemble conveys mechanistic information about the underlying gene.

### Comparison to related methods

A number of analytical results map CDRCs or their ratios to protein distribution shape [17], [22], [25], [28], [38]–[40]. However, only Friedman, Cai and Xie's work demonstrating that protein counts are gamma-distributed [25] has been co-opted for solving the inverse problem: using distribution shape to infer the parameters [42]–[44]. Their result allows one to directly compute 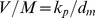 and 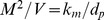 from a gamma distribution's measured mean (M) and variance (V). We compared ACES' performance estimating the same ratios in the same regime as the one assumed by the Friedman model.

First, we screened our library for CDRC input sets where 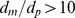, average protein count >1000, and 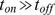 (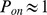). These restrictions correspond to the Friedman assumptions that 1) 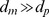, 2) one can use a continuous approximation for protein count, and 3) the gene is always active. 251 input sets (4%) in our library satisfy these criteria. We examined the output solutions ACES returned for these specific input sets. Because ACES makes no assumptions about parameter regime, it returns solutions that conform to the Friedman assumptions as well as many additional solutions that do not. To compare ACES with the Friedman approach on equal footing, we first only considered ACES solutions that were consistent with the Friedman criteria. This set of solutions accurately identifies both Friedman ratios; the solutions cluster exactly on the true solution. By relative error, the ACES solution slightly outperforms Friedman's analytical estimation of both ratios (Fig. S11A,B): for 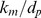, the median relative error in the estimate was .024, versus a median error of .076 for the Friedman estimator. In addition to accurately recovering the Friedman ratios, ACES also recovered other CDRCs or ratios not obtainable from the Friedman model (Fig. S10B). Average RNA count was the most commonly identified parameter besides 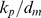 and 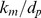. This exercise serves as a control for our method, demonstrating equivalence between our method and the Friedman analytical solution, in the regime assumed by the Friedman model.

However, ACES' utility derives from not assuming any particular parameter regime. We therefore compared the solutions that did not conform to the Friedman regime to the solutions that did conform. Even the solutions that did not conform to the Friedman assumption are highly enriched for successful fitting of the Friedman ratios 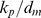 and 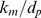. Of 251 input sets, 222 have an MD<.75. Both ratios share an average MD value of .33, as compared to the overall library MD average of 1.25. Of the 29 input sets for which these ratios were fit with an MD >.75, all of them demonstrate bimodality in histograms of both ratios, suggesting that solutions outside the Friedman regime can produce gamma distributed protein distributions. The value of ACES is demonstrated by its ability to accurately fit the Friedman ratios even in regimes that explicitly violate the Friedman assumptions.

We explored this point further and discovered that the model of gene expression studied in this report (Fig. 1) readily generates gamma distributions, most of which do not correspond to the Friedman regime. There were 995 CDRC sets in our library that generate gamma distributed protein distributions (See examples, Fig. S11C,D). Of these, only 346 demonstrate accurate inference of the 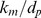 and 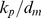 ratios using the Friedman model. In Fig. S11E, ACES' fit of the distribution is compared to the Friedman fit (magenta triangles), revealing that while ACES' solution ensemble contains the correct answer, the Friedman solution settles on the incorrect value for both ratios. These results emphasize that having a gamma distribution is necessary but not sufficient for fitting the Friedman ratios, and reinforce that ACES operates robustly outside of the Friedman regime.

## Discussion

Protein count distributions are readily measured from clonal populations of reporter-gene bearing cells [11], [54], yet we lack a reliable computational framework for abstracting molecular mechanisms from distribution shape. Here we presented an efficient, assumption-free approach to inferring molecular mechanism from protein distribution shape. Though the full analytical solution of the protein distribution remains elusive, we were able to solve for exact expressions of the distributions' higher order moments, extending previous work which stopped at variance [5], [9], [10], [13], [15], [25], [26], [28], [39], [45]. We found that the higher order moments skewness and kurtosis contributed significantly to the fitting problem; indeed, when fitting only on the mean and variance, we were rarely able to identify a CDRC or CDRC ratio in our representative library of test sets (column MV, Table 1).

We found that four moments accurately reproduce the complete protein distribution, even in the worst case scenario when very different CDRC sets map to the same four moments (Figs. 3, S2–S6). This is particularly exciting given the challenges of solving anything but the simplest chemical master equation model, as the moment-matching approach presented herein, while inelegant, is perfectly general. Even in cases where moments do not exactly capture distribution shape, moment matching could allow investigators to rule out the vast majority of candidate solutions, limiting the use of computationally intensive Gillespie simulations to cases where a given parameter set is likely to be correct.

ACES, our algorithm for linking parameters to moments, proved to be efficient enough to identify *all* CDRC sets consistent with each of 8053 input sets. To build confidence in our results, we checked whether ACES' CDRC ensembles agreed with a previous analytical result, which states that in a specific regime, gene expression is gamma distributed and the ratios 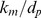 and 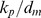 are directly calculable from the mean and variance [25]. We found that when limited to this regime, ACES' not only agreed with the Friedman results, but also frequently identified other expression-related quantities, such as the average RNA expression level, 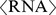 and 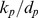. In many cases, assuming the Friedman regime allowed direct inference of most of the remaining CDRCs and ratios (compare Fig. S10A to S10B), quantities which cannot be obtained by the Friedman model.

CDRC sets operating in the Friedman regime represented only 4% of the possible regimes we studied in our library, reinforcing the importance of bringing to bear an unrestricted framework for analyzing protein distributions. Opening our analysis to the whole library, we discovered several trends. First, about one in eight regimes recapitulate a gamma distribution, but only about one in three of gamma distributed input sets fall in the Friedman regime. In other words, model agreement with a distribution does not prove model validity. This recalls a similar outcome from Zopf *et al*, where the standard ON-OFF model studied here (Fig. 1) accurately models total population distributions, but fails to capture subpopulation distributions partitioned by cell cycle phase [21]. Both results provide motivation for continued refinement of stochastic gene expression models, which will benefit from the parameter estimation approach suggested in this work.

Second, we find that the exact nature of degeneracy in a solution set informs on mechanisms that underlie the shape of the corresponding protein distribution. Every library member corresponds to a unique ensemble of solutions. Sometimes, solution histograms encompass the complete range of a given CDRC or CDRC ratio, while often the range of a parameter is confined to a fraction of its possible space. Sometimes ACES reveals the only CDRC or CDRC ratio value consistent with a distributions shape. All results are informative. In some cases, ACES revealed bimodality in a particular parameter, suggesting that the degeneracy arises from uncertainty in one particular CDRC (Fig. 5). In other examples, we found that ACES reveals burst histograms consistent with slow ON-OFF transitioning behavior, even without always exactly identifying the burst parameter (Fig. S9). Even knowing when a distribution cannot rule out any value of a CDRC parameter or CDRC ratio, as in Fig. S7, informs an investigator that additional information is needed to further constrain the fitting problem.

Third, some ratios and CDRCs are more readily abstracted from the distribution than others. While the ON-OFF transition CDRCs were particularly challenging to infer, especially 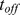, we found that 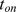 and 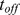 pairs often varied while conserving one value of 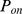. If this observation bears out in experimental validation, accessibility to 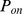 suggests a novel way to parameterize another class of models, the so-called thermodynamic model of *cis*-regulation [55]–[57]. At the core of a thermodynamic model is a partition function that divides transcriptionally active promoter states by all molecular states, and is equal to 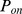. Though ACES readily identified a variety of CDRC ratios besides 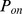 we were surprised to find individual CDRC values fit, despite evidence that CDRCs cannot be obtained from steady state distributions alone [41]. We speculate that explicitly confining the search to the physiological domain of each parameter permitted estimation of some CDRCs. Importantly, bounding CDRCs but fitting only on mean and variance did not permit much CDRC estimation, indicating that it is the full protein distribution in conjunction with parameter ranges that enables ACES to estimate individual CDRCs. Independent of CDRC or ratio, further constraining ACES with measures of one or both of the degradation rates naturally improves parameter estimation across the board (Table 1).

Although we explored CDRC estimation only when restricting 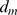 and 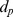, the algorithm accepts user-defined constraints accounting for uncertainty in both the CDRCs and the moments. For example, an investigator may have experimentally determined the 95% confidence interval for a particular parameter. Confining the ACES search to this interval requires only replacing the physiological range with measured bounds. Similarly, measurement of distribution moments will be imperfect, likely with higher moments admitting more significant error. Again, one can adjust ACES' tolerance for each moment. This flexibility means that the worst outcome manifests as CDRC values spanning their entire range; an investigator need not fear overfitting or spurious convergence.

More importantly perhaps, ACES fits will aid investigators in model selection. ACES fit outcomes fall into three categories: (1) ACES fails to discover a candidate CDRC set (2) ACES finds CDRC solutions spanning most or all of the range of each parameter, or (3) ACES narrows down some or all CDRCs to a limited range. As discussed above, high experimental error or not enough constraints can lead to outcome (2). Both (1) and (3) are potentially instructive regarding model selection. In (1), either moment error tolerance was more strict than measurement error, ACES was not run with a sufficiently high enough resolution, or the model does not adequately explain the data. The first two possibilities are easily ruled out, and if excluded indicate the ON-OFF model insufficiently characterizes a particular set of moments, and therefore the distribution. Several examples in the literature highlight alternative models, including the possibility of a refractory OFF state [8], multiple ON states [30], and even transcription rates that depend on the cell cycle [21]. This last example illustrates how cellular state, called extrinsic noise [5], [14], [27], intertwines with the intrinsic-only stochasticity captured by our approach. While a variety of methods have been developed for measuring and minimizing the impact of extrinsic noise [11], [12], [58]–[60], Zopf *et al* reinforce the idea that extrinsic can be made intrinsic by explicitly incorporating fluctuating inputs into a given model [21], [24], [29], [60]. Though we focused solely on the ON-OFF model, the paradigm suggested here of solving for and exhaustively fitting to moments is perfectly viable for any linear stochastic model, including those that admit fluctuating inputs or comprise more elaborate promoter architectures. Whether the additional CDRCs implicit to more detailed models can be inferred from stationary distributions is an open question. The significant degeneracy we encountered with the ON-OFF model in this work suggests that significant constraints will be necessary for such distributions to be informative.

Having some CDRCs or CDRC ratios well fit while others span wider ranges, as in (3), suggest either the data are not sufficiently constrained to distinguish each parameter, or the model is too complex. We saw this latter scenario in our data; a variety of choices of 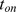 , 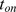 , and 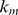 can conspire to drive transcription as a Poisson process at rate 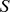. However, by testing for constraint of various CDRC ratios, these patterns arise naturally in our framework. We saw numerous examples of model reduction. Encouragingly, we rediscovered the Friedman solution, a two parameter model characterized by 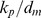 and 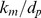 [25]. Their parameterization posits protein production as the product of the number of transcripts produced per cell division (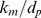), assuming that dilution dominates protein loss, and the number of proteins produced in the lifetime of an RNA (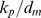). In addition, we found alternate two-parameter formulations, for example, 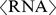 and 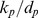 , suggesting that in some regimes this pair of ratios ascribes shape to the distribution, while the Friedman ratios do not. Of a large number of test sets where 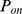 was fit, 387 cases identified *only* 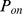. Though we did not directly interrogate the ratio 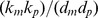, fitting 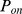 and given the protein mean guarantees that this four-CDRC ratio is also fit in those test sets, providing yet another two parameter model. Thus, the only undesirable consequence of assuming a more complex model is increased computational time, a potential roadblock that we did not encounter in the current study. Indeed, the more general model collapses into an intriguing number of unique simpler models that might otherwise not be intuited by an investigator (Data S1).

ACES will enable a more mechanistic understanding of gene regulation. We envision it will be most informative when used in pairwise studies of reporter gene activity, for example, by measuring reporter gene distributions before and after knocking out a histone modifying enzyme, or a transcription factor binding site in a promoter. Historically these manipulations reveal only whether a *cis-* or *trans-* acting factor activates or represses gene activity. By incorporating data from changes in stochasticity, we expect to refine our understanding of regulation by ascertaining which CDRC, or set of CDRCs, are regulated by a particular sequence element or protein. Importantly, even if the exact values of CDRCs cannot be obtained from a distribution, changes in the range of a particular parameter still provide mechanistic insight into the effects of specific genetic perturbations. Previous studies incorporating noise into their analysis reveal insights such as these, for example, that the mammalian *cis*-regulatory CCAAT box element chiefly modulates the 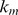 and 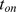 CDRCs, but has little impact on 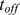 [8].

Given that four moments capture the full shape of the protein expression distribution, and that we can rapidly determine all CDRC sets consistent with these moments, this approach shows great promise in learning mechanistic information from stochasticity in protein expression. We applied our framework to one particular steady state protein distribution (Fig. 1), but the general approach is amenable to analyzing any first-order chemical master equation model of protein or RNA distributions, RNA-protein joint distributions, and even distributions evolving over time. We expect the application of ACES to improve our understanding of the fundamental processes that govern gene expression.

## Materials and Methods

### ACES algorithm overview

For the first parameter 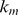, we select its first value, for example, 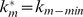. Given the mean expression level from a population of cells (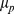), the equation for how this mean relates to the CDRCs,
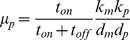
(5)and the CDRC minima and maxima, [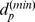, 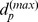], [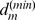, 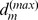], [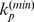, 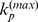], [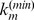, 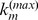], [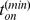, 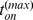], [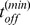, 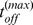], we can potentially further constrain the boundaries on the next CDRC to be checked. If the next parameter to be selected is 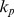, then we can check the boundaries on 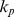 given all the information we have to this point. That is, the new 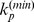 might be greater than the physiological 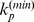 given that 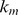 is fixed at 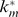, and the mean expression level is fixed at 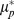. Substituting in the known values up to this point into Eq. 5, we get:
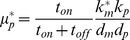
(6)


Then solving for 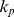 we get:
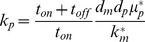
(7)


The result is a monotonic function for all variables; thus, 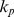
's minimum and maximum values can be obtained, subject to the fixed parameters to this point (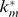 and 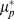) and the physiological minima and maxima of the remaining free parameters:
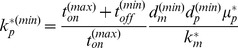
(8)

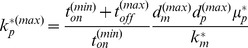
(9)


These candidate minimum and maximum values are not guaranteed to be greater than or less than, respectively, the physiologically determined bounds for 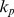. To take this into account, we accept 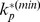 if it is greater than 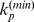, and we accept 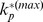 if it is less than 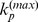. Thus the new boundaries define a range for 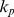 that is less than or equal to the physiological range defined at runtime. In the next step, the algorithm subdivides the new 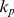 range with resolution 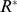, shrinking the resolution 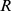 by the fraction of parameter space lost by accepting a narrower CDRC range. The algorithm then selects the first value of 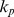 in this new range, and repeats the steps just outlined above with the next parameter.

Because the mean equation is linear, at the end of this process the last parameter can be directly solved for. At that point, we have a set of CDRCs 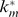, 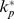, 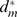, 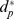, 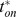, and 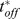 which, by the nature of how each parameter was selected, already exactly equal the first moment equation (Eq. 5). We then plug in this candidate CDRC set into our variance solution; if the calculated variance also matches within 1% of the measured variance, we test skewness with the same criteria, and then again with kurtosis. If the 2nd through 4th moments all agree with the input (measured) moments, this CDRC set is recorded as a solution.

The full pseudocode is provided in the Supplement, and an implementation of the algorithm is also provided in C++ (Data S2).

### Log Euclidean distance

Log Euclidean distances are computed as follows. For moment space, log-euclidean distance between two moment sets 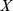 and 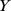, with elements mean (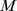), variance (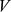), skewness (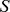), and kurtosis (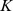) would be:
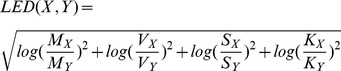
(10)


For CDRCs, the equation would be logs of ratios of CDRCs rather than moments, and there would be six summands rather than four. We used log distance so that a CDRC–or a moment–changing from .01 to .1 contributes the same weight as another parameter changing from 100 to 1000. Thus, very different scale parameters, or moments, can be compared.

### Jensen-Shannon Divergence

Jensen-Shannon Divergence for probability distributions A and B (JSD(A,B)) is defined as
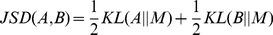
(11)given the definitions for Kullback-Leibler divergence (KL) and the mixture distribution (M)
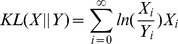
(12)

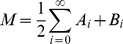
(13)


## Supporting Information

Data S1 **ACES library data.** ACES was run for each of the 8053 library members, generating a list of solutions. The summary data for each ACES run is described is included in the excel spreadsheet. Data S1 is the source of the library summary results reported in Table 1. A more detailed explanation of each datasheet and column can be found in the README datasheet within Data S1.
(XLSX)

Data S2 **ACES algorithm and moment solution routines.** This archive contains both the ACES algorithm routine written in C++ and a Matlab implementation of the moment solutions. See README.txt for more detail.
(ZIP)

Text S1 **Supporting information.** This supplement documents the moments derivation and validation, continues the explanation of the ACES algorithm, and contains the supporting figures referenced throughout the text.
(PDF)
