# Supplementary material for: A Computational Framework for Analyzing Stochasticity in Gene Expression
Source: PLoS Comput Biol. 2014 May 8;10(5):e1003596. doi: 10.1371/journal.pcbi.1003596 (PMC4014403; doi:10.1371/journal.pcbi.1003596)
Supplement: Text S1 — Supporting information. This supplement documents the moments derivation and validation, continues the explanation of the ACES algorithm, and contains the supporting figures referenced throughout the text. (PDF) [file pcbi.1003596.s003.pdf]

Supplemental Information. A framework for inferring rate constants from stochasticity in gene expression

Marc S. Sherman[1,2], Barak A. Cohen[2,*]

1 Computational and Molecular Biophysics, Washington University in St. Louis, St. Louis, MO, United States

2 Center for Genome Sciences, Department of Genetics, Washington University in St. Louis, St. Louis, MO, United States

*** E-mail: cohen@genetics.wustl.edu**

Contents

S1 Analytical expressions for the steady state central moments **S2**

S2 Validation of moment equations **S10**

S3 Analytically Constrained Exhaustive Search (ACES) **S11**

S3.1 Algorithm S11

S3.2 Reverse order algorithm S14

S4 CDRC input library **S15**

S5 Contribution of the 5th moment **S16**

S6 Gillespie simulations **S17**

S7 Identifying gamma distributions across the library **S17**

S1 Analytical expressions for the steady state central moments

For convenience, the CME equations for the general ON-OFF model of gene expression shown in the main text are reproduced here.

$$\begin{aligned}\frac{dP_0(m,q)}{dt} =& t_{off}P_1(m,q) + d_m(m+1)P_0(m+1,q) \\ &+ k_p m P_0(m,q-1) + d_p(q+1)P_0(m,q+1) \\ &- (k_m + md_m + mk_p + d_p q + t_{on})P_0(m,q)\end{aligned} \tag{S1}$$

$$\begin{aligned}\frac{dP_1(m,q)}{dt} =& t_{on}P_0(m,q) + d_m(m+1)P_1(m+1,q) \\ &+ k_p m P_1(m,q-1) + d_p(q+1)P_1(m,q+1) \\ &+ k_m P_1(m-1,q) \\ &- (k_m + md_m + mk_p + d_p q + t_{off})P_1(m,q)\end{aligned} \tag{S2}$$

For simplicity we separated the CME into two parallel equations; one corresponding to the probabilities of m RNAs and q proteins when the gene is in the ON state, and one corresponding to the same probabilities when the gene is in the OFF state.

To derive the generic i-th RNA moment and j-th protein moment, we multiplied both sides of Eq. 1 and 2 by $m^i q^j$, then summed across all m and q, as in Sanchez and Kondev [1]. For clarity we will perform the following algebra on a parameter by parameter basis.

For t_{on} and the LHS in Eq. 2,

$$\sum_{m_1=0}^{\infty}\sum_{q_1=0}^{\infty} m_1^i q_1^j \frac{dP_1(m_1,q_1)}{dt} = \sum_{m_1=0}^{\infty}\sum_{q_1=0}^{\infty} m_1^i q_1^j t_{on} P_0(m_0,q_0) \tag{S3}$$

$$\frac{d\langle m_1^i q_1^j\rangle}{dt} = t_{on}\langle m_0^i q_0^j\rangle + ... \tag{S4}$$

where the moments are derived from the identity that the i-th moment of n is equal to $\sum_{n=0}^{\infty} n^i p(n)$. The subscripts above, e.g., $\langle m_0^i q_0^j\rangle$, correspond to the partial moments with respect to the promoter state. Since non-central moments are additive, the sum of partial moments across all promoter states equals

the complete moment.

$$\langle m^i q^j \rangle = \langle m_0^i q_0^j \rangle + \langle m_1^i q_1^j \rangle \tag{S5}$$

Continuing the derivation of Eq. 4 in the main text, piecewise, for d_m we have,

$$... + \sum_{m_1=0}^{\infty} \sum_{q_1=0}^{\infty} m_1^i q_1^j d_m((m_1+1)P_1(m_1+1,q_1) - m_1 P_1(m_1,q_1)) + ... \tag{S6}$$

$$... + \sum_{m_1=0}^{\infty} \sum_{q_1=0}^{\infty} d_m(m_1^i q_1^j (m_1+1)P_1(m_1+1,q_1) - m_1^{i+1} q_1^j P_1(m_1,q_1)) + ... \tag{S7}$$

$$... + d_m(\sum_{m_1=1}^{\infty} \sum_{q_1=0}^{\infty} ((m_1-1)^i q_1^j m_1 P_1(m_1,q_1) - \sum_{m_1=0}^{\infty} \sum_{q_1=0}^{\infty} m_1^{i+1} q_1^j P_1(m_1,q_1))) + ... \tag{S8}$$

$$... + d_m(\sum_{m_1=1}^{\infty} \sum_{q_1=0}^{\infty} ((m_1-1)^i q_1^j m_1 - m_1^{i+1} q_1^j) P_1(m_1,q_1)) + ... \tag{S9}$$

$$... + d_m \langle m_1(m_1-1)^i q_1^j + m_1^{i+1} q_1^j \rangle + ... \tag{S10}$$

Note from line 7 to 8 we change the summation across RNA in order to remove the m_1+1 term inside $P_1(m_1+1, q_1)$ term. Then, noting for the second summation that when $m = 0$ the expression also goes to zero, we re-unite both sides with the summation across m starting at 1. We use this same manipulation in future steps.

For k_m,

$$... + \sum_{m_1=0}^{\infty} \sum_{q_1=0}^{\infty} m_1^i q_1^j k_m(P_1(m_1-1,q_1) - P_1(m_1,q_1)) + ... \tag{S11}$$

$$... + k_m(\sum_{m_1=0}^{\infty} \sum_{q_1=0}^{\infty} m_1^i q_1^j P_1(m_1-1,q_1) - m_1^i q_1^j P_1(m_1,q_1)) + ... \tag{S12}$$

$$... + k_m(\sum_{m_1=-1}^{\infty} \sum_{q_1=0}^{\infty} (m_1+1)^i q_1^j P_1(m_1,q_1) - \sum_{m_1=0}^{\infty} \sum_{q_1=0}^{\infty} m_1^i q_1^j P_1(m_1,q_1)) + ... \tag{S13}$$

$$... + k_m(\sum_{m_1=0}^{\infty} \sum_{q_1=0}^{\infty} ((m_1+1)^i q_1^j - m_1^i q_1^j) P_1(m_1,q_1)) + ... \tag{S14}$$

$$... + k_m \langle (m_1+1)^i q_1^j - m_1^i q_1^j \rangle + ... \tag{S15}$$

Line 15 is exactly true for all $j \neq 0$. When $j = 0$, any choice of i will produce a polynomial of moments plus a constant $\langle 1 \rangle$. Since $\langle 1 \rangle$ corresponds to the following underlying summation, we recognize this is simply the amount of probability mass contained in the ON state.

$$\sum_{m_1=0}^{\infty} \sum_{q_1=0}^{\infty} P_1(m_1, q_1) = \frac{t_{on}}{t_{on} + t_{off}} \tag{S16}$$

For k_p,

$$... + k_p(\sum_{m_1=0}^{\infty} \sum_{q_1=0}^{\infty} m_1^{i+1} q_1^j P_1(m_1, q_1 - 1) - m_1^{i+1} q_1^j P_1(m_1, q_1)) + ... \tag{S17}$$

$$... + k_p(\sum_{m_1=0}^{\infty} \sum_{q_1=-1}^{\infty} m_1^{i+1} (q_1 + 1)^j P_1(m_1, q_1) - \sum_{m_1=0}^{\infty} \sum_{q_1=0}^{\infty} m_1^{i+1} q_1^j P_1(m_1, q_1)) + ... \tag{S18}$$

$$... + k_p(\sum_{m_1=0}^{\infty} \sum_{q_1=0}^{\infty} (m_1^{i+1} (q_1 + 1)^j - m_1^{i+1} q_1^j) P_1(m_1, q_1)) + ... \tag{S19}$$

$$... + k_p \langle m_1^{i+1} (q_1 + 1)^j - m_1^{i+1} q_1^j \rangle + ... \tag{S20}$$

For d_p,

$$... + d_p(\sum_{m_1=0}^{\infty} \sum_{q_1=0}^{\infty} m_1^i q_1^j (q_1 + 1) P_1(m_1, q_1 + 1) - m_1^i q_1^{j+1} P_1(m_1, q_1)) + ... \tag{S21}$$

$$... + d_p(\sum_{m_1=0}^{\infty} \sum_{q_1=1}^{\infty} m_1^i (q_1 - 1)^j q_1 P_1(m_1, q_1) - \sum_{m_1=0}^{\infty} \sum_{q_1=0}^{\infty} m_1^i q_1^{j+1} P_1(m_1, q_1)) + ... \tag{S22}$$

$$... + d_p(\sum_{m_1=0}^{\infty} \sum_{q_1=0}^{\infty} (m_1^i (q_1 - 1)^j q_1 - m_1^i q_1^{j+1}) P_1(m_1, q_1)) + ... \tag{S23}$$

$$... + d_p \langle m_1^i (q_1 (q_1 - 1)^j - q_1^{j+1}) \rangle + ... \tag{S24}$$

Lastly, for t_{off},

$$... - \sum_{m_1=0}^{\infty}\sum_{q_1=0}^{\infty} m_1^i q_1^j t_{off} P_1(m_1, q_1) \tag{S25}$$

$$... - t_{off}\langle m_1^i q_1^j \rangle \tag{S26}$$

Combining Eqs. 4, 10, 15, 20, 24, and 26, we get Eq. 4 from the main body, and reproduced here:

$$\begin{aligned} \frac{d\langle m_1^i q_1^j\rangle}{dt} =& t_{on}\langle m_0^i q_0^j\rangle - t_{off}\langle m_1^i q_1^j\rangle \\ &+ k_m\langle (m_1+1)^i q_1^j - m_1^i q_1^j\rangle \\ &+ d_m\langle m_1(m_1-1)^i q_1^j + m_1^{i+1} q_1^j\rangle \\ &+ k_p\langle m_1^{i+1}(q_1+1)^j - m_1^{i+1} q_1^j\rangle \\ &+ d_p\langle m_1^i (q_1(q_1-1)^j - q_1^{j+1})\rangle \end{aligned} \tag{S27}$$

Following identical procedures but starting with Eq. S1 we arrive at the second partial moment expansion (Eq. 3 in the main text):

$$\begin{aligned} \frac{d\langle m_0^i q_0^j\rangle}{dt} =& t_{off}\langle m_1^i q_1^j\rangle - t_{on}\langle m_0^i q_0^j\rangle \\ &+ d_m\langle m_0(m_0-1)^i q_0^j + m_0^{i+1} q_0^j\rangle \\ &+ k_p\langle m_0^{i+1}(q_0+1)^j - m_0^{i+1} q_0^j\rangle \\ &+ d_p\langle m_0^i (q_0(q_0-1)^j - q_0^{j+1})\rangle \end{aligned} \tag{S28}$$

To generate the partial moment ODEs we then plugged in the following values of i and j : for integer $n = 0$ to 4, we compute all integer moments that satisfy $i + j = n$ for $i, j \geq 0$. For example, when $n = 3$, we compute the following pairs: $(i = 0, j = 3), (i = 1, j = 2), (i = 2, j = 1), (i = 3, j = 0)$. The result is 14 combinations of i and j for each promoter state, or 28 partial moment ODE equations.

For n=1,

$$\frac{d\langle m_1\rangle}{dt} = k_m \frac{t_{on}}{t_{on}+t_{off}} + t_{on}\langle m_0\rangle - t_{off}\langle m_1\rangle - d_m\langle m_1\rangle \tag{S29}$$

$$\frac{d\langle m_0\rangle}{dt} = t_{off}\langle m_1\rangle - t_{on}\langle m_0\rangle - d_m\langle m_0\rangle \tag{S30}$$

$$\frac{d\langle q_1\rangle}{dt} = k_p\langle m_1\rangle + t_{on}\langle q_0\rangle - d_p\langle q_1\rangle - t_{off}\langle q_1\rangle \tag{S31}$$

$$\frac{d\langle q_0\rangle}{dt} = k_p\langle m_0\rangle + t_{off}\langle q_1\rangle - d_p\langle q_0\rangle - t_{on}\langle q_0\rangle \tag{S32}$$

For n=2,

$$\frac{d\langle m_1^2\rangle}{dt} = t_{on}\langle m_0^2\rangle + 2k_m\langle m_1\rangle + k_m \frac{t_{on}}{t_{on}+t_{off}} - t_{off}\langle m_1^2\rangle - 2d_m\langle m_1^2\rangle + d_m\langle m_1\rangle \tag{S33}$$

$$\frac{d\langle m_0^2\rangle}{dt} = t_{off}\langle m_1^2\rangle - t_{on}\langle m_0^2\rangle - 2d_m\langle m_0^2\rangle + d_m\langle m_0\rangle \tag{S34}$$

$$\frac{d\langle q_1^2\rangle}{dt} = t_{on}\langle q_0^2\rangle + d_p\langle q_1\rangle + k_p\langle m_1\rangle + 2k_p\langle q_1 m_1\rangle - (2d_p + t_{off})\langle q_1^2\rangle \tag{S35}$$

$$\frac{d\langle q_0^2\rangle}{dt} = t_{off}\langle q_1^2\rangle + d_p\langle q_0\rangle + k_p\langle m_0\rangle + 2k_p\langle q_0 m_0\rangle - (2d_p + t_{on})\langle q_0^2\rangle \tag{S36}$$

$$\frac{d\langle m_1 q_1\rangle}{dt} = t_{on}\langle q_0 m_0\rangle - t_{off}\langle q_1 m_1\rangle - d_m\langle q_1 m_1\rangle - d_p\langle q_1 m_1\rangle + k_m\langle q_1\rangle + k_p\langle m_1^2\rangle \tag{S37}$$

$$\frac{d\langle m_0 q_0\rangle}{dt} = t_{off}\langle q_1 m_1\rangle - t_{on}\langle q_0 m_0\rangle - d_m\langle q_0 m_0\rangle - d_p\langle q_0 m_0\rangle + k_p\langle m_0^2\rangle \tag{S38}$$

For n=3,

$$\frac{d\langle m_1^3\rangle}{dt} = t_{on}\langle m_0^3\rangle - t_{off}\langle m_1^3\rangle + 3k_m\langle m_1^2\rangle + 3k_m\langle m_1\rangle + k_m\frac{t_{on}}{t_{on}+t_{off}} - d_m(3(\langle m_1^3\rangle - \langle m_1^2\rangle) + \langle m_1\rangle) \tag{S39}$$

$$\frac{d\langle m_0^3\rangle}{dt} = t_{off}\langle m_1^3\rangle - t_{on}\langle m_0^3\rangle - d_m(3(\langle m_0^3\rangle - \langle m_0^2\rangle) + \langle m_0\rangle) \tag{S40}$$

$$\frac{d\langle q_1^3\rangle}{dt} = t_{on}\langle q_0^3\rangle - t_{off}\langle q_1^3\rangle + k_p\langle m_1\rangle + 3k_p\langle q_1 m_1\rangle + 3k_p\langle q_1^2 m_1\rangle + 3d_p\langle q_1^2\rangle - d_p\langle q_1\rangle - 3d_p\langle q_1^3\rangle \tag{S41}$$

$$\frac{d\langle q_0^3\rangle}{dt} = t_{off}\langle q_1^3\rangle - t_{on}\langle q_0^3\rangle + k_p\langle m_0\rangle + 3k_p\langle q_0 m_0\rangle + 3k_p\langle q_0^2 m_0\rangle + 3d_p\langle q_0^2\rangle - d_p\langle q_0\rangle - 3d_p\langle q_0^3\rangle \tag{S42}$$

$$\frac{d\langle q_1^2 m_1\rangle}{dt} = t_{on}\langle q_0^2 m_0\rangle - t_{off}\langle q_1^2 m_1\rangle + k_m\langle q_1^2\rangle - d_m\langle q_1^2 m_1\rangle + k_p\langle m_1^2\rangle + 2k_p\langle q_1 m_1^2\rangle + d_p\langle q_1 m_1\rangle - 2d_p\langle q_1^2 m_1\rangle \tag{S43}$$

$$\frac{d\langle q_0^2 m_0\rangle}{dt} = t_{off}\langle q_1^2 m_1\rangle - t_{on}\langle q_0^2 m_0\rangle - d_m\langle q_0^2 m_0\rangle + k_p\langle m_0^2\rangle + 2k_p\langle q_0 m_0^2\rangle + d_p\langle q_0 m_0\rangle - 2d_p\langle q_0^2 m_0\rangle \tag{S44}$$

$$\frac{d\langle q_1 m_1^2\rangle}{dt} = t_{on}\langle q_0 m_0^2\rangle - t_{off}\langle q_1 m_1^2\rangle + k_p\langle m_1^3\rangle + k_m\langle q_1\rangle + (2k_m + d_m)\langle q_1 m_1\rangle - (2d_m + d_p)\langle q_1 m_1^2\rangle \tag{S45}$$

$$\frac{d\langle q_0 m_0^2\rangle}{dt} = t_{off}\langle q_1 m_1^2\rangle - t_{on}\langle q_0 m_0^2\rangle + k_p\langle m_0^3\rangle + d_m\langle q_0 m_0\rangle - (2d_m + d_p)\langle q_0 m_0^2\rangle \tag{S46}$$

For n=4,

$$\frac{d\langle m_1^4\rangle}{dt} = t_{on}\langle m_0^4\rangle - t_{off}\langle m_1^4\rangle + k_m\frac{t_{on}}{t_{on}+t_{off}} + 4\langle m_1\rangle + 6\langle m_1^2\rangle + 4\langle m_1^3\rangle) + d_m(\langle m_1\rangle - 4\langle m_1^2\rangle + 6\langle m_1^3\rangle - 4\langle m_1^4\rangle) \tag{S47}$$

$$\frac{d\langle m_0^4\rangle}{dt} = t_{off}\langle m_1^4\rangle - t_{on}\langle m_0^4\rangle + d_m(\langle m_0\rangle - 4\langle m_0^2\rangle + 6\langle m_0^3\rangle - 4\langle m_0^4\rangle) \tag{S48}$$

$$\frac{d\langle q_1^4\rangle}{dt} = t_{on}\langle q_0^4\rangle - t_{off}\langle q_1^4\rangle + k_p(\langle m_1\rangle + 4\langle q_1m_1\rangle + 6\langle q_1^2m_1\rangle + 4\langle q_1^3m_1\rangle) + d_p(\langle q_1\rangle - 4\langle q_1^2\rangle + 6\langle q_1^3\rangle - 4\langle q_1^4\rangle) \tag{S49}$$

$$\frac{d\langle q_0^4\rangle}{dt} = t_{off}\langle q_1^4\rangle - t_{on}\langle q_0^4\rangle + k_p(\langle m_0\rangle + 4\langle q_0m_0\rangle + 6\langle q_0^2m_0\rangle + 4\langle q_0^3m_0\rangle) + d_p(\langle q_0\rangle - 4\langle q_0^2\rangle + 6\langle q_0^3\rangle - 4\langle q_0^4\rangle) \tag{S50}$$

$$\frac{d\langle q_1^3m_1\rangle}{dt} = t_{on}\langle q_0^3m_0\rangle - t_{off}\langle q_1^3m_1\rangle + k_m\langle q_1^3\rangle - d_m\langle q_1^3m_1\rangle + k_p(\langle m_1^2\rangle + 3\langle q_1m_1^2\rangle + 3\langle q_1^2m_1^2\rangle) - d_p(\langle q_1m_1\rangle - 3\langle q_1^2m_1\rangle + 3\langle q_1^3m_1\rangle) \tag{S51}$$

$$\frac{d\langle q_0^3m_0\rangle}{dt} = t_{off}\langle q_1^3m_1\rangle - t_{on}\langle q_0^3m_0\rangle - d_m\langle q_0^3m_0\rangle + k_p(\langle m_0^2\rangle + 3\langle q_0m_0^2\rangle + 3\langle q_0^2m_0^2\rangle) - d_p(\langle q_0m_0\rangle - 3\langle q_0^2m_0\rangle + 3\langle q_0^3m_0\rangle) \tag{S52}$$

$$\frac{d\langle q_1^2m_1^2\rangle}{dt} = t_{on}\langle q_0^2m_0^2\rangle - t_{off}\langle q_1^2m_1^2\rangle + k_m(\langle q_1^2\rangle + 2\langle q_1^2m_1\rangle) + d_m(\langle q_1^2m_1\rangle - 2\langle q_1^2m_1^2\rangle) + k_p(\langle m_1^3\rangle + 2\langle q_1m_1^3\rangle) + d_p(\langle q_1m_1^2\rangle - 2\langle q_1^2m_1^2\rangle) \tag{S53}$$

$$\frac{d\langle q_0^2m_0^2\rangle}{dt} = t_{off}\langle q_1^2m_1^2\rangle - t_{on}\langle q_0^2m_0^2\rangle + d_m(\langle q_0^2m_0\rangle - 2\langle q_0^2m_0^2\rangle) + k_p(\langle m_0^3\rangle + 2\langle q_0m_0^3\rangle) + d_p(\langle q_0m_0^2\rangle - 2\langle q_0^2m_0^2\rangle) \tag{S54}$$

$$\frac{d\langle q_1m_1^3\rangle}{dt} = t_{on}\langle q_0m_0^3\rangle - t_{off}\langle q_1m_1^3\rangle + k_m(\langle q_1\rangle + 3\langle q_1m_1\rangle + 3\langle q_1m_1^2\rangle) - d_m(\langle q_1m_1\rangle - 3\langle q_1m_1^2\rangle + 3\langle q_1m_1^3\rangle) + k_p\langle m_1^4\rangle - d_p\langle q_1m_1^3\rangle \tag{S55}$$

$$\frac{d\langle q_0m_0^3\rangle}{dt} = t_{off}\langle q_1m_1^3\rangle - t_{on}\langle q_0m_0^3\rangle - d_m(\langle q_0m_0\rangle - 3\langle q_0m_0^2\rangle + 3\langle q_0m_0^3\rangle) + k_p\langle m_0^4\rangle - d_p\langle q_0m_0^3\rangle \tag{S56}$$

We then set each of the partial moment derivatives (LHS) to zero and solved for the partial moment terms in Mathematica (Wolfram Research, Champaign, IL, 2010). There were 28 unknown partial moments and 28 equations, and we were able to solve exactly for each one in terms of the 6 CDRCs, t_{on}, t_{off}, k_m, d_m, k_p, and d_p. From these we computed the 14 moments about zero from the partial moments

using Eq. 5.

We examined the mean plus the next 3 non-normalized central moments. The j-th central moment μ_j is defined as

$$\mu_j = \langle (q - \langle q \rangle)^j \rangle \tag{S57}$$

Expansions of μ_j for $j = 2, 3, 4$ are as follows:

$$\mu_2 = \langle q^2 \rangle - \langle q \rangle^2 \tag{S58}$$

$$\mu_3 = \langle q^3 \rangle - 3\langle q \rangle \langle q^2 \rangle + 2\langle q \rangle^3 \tag{S59}$$

$$\mu_4 = \langle q^4 \rangle - 4\langle q^3 \rangle \langle q \rangle + 6\langle q^2 \rangle \langle q \rangle^2 - 3\langle q \rangle^4 \tag{S60}$$

where the moments about zero $\langle q^j \rangle$ are exactly what we computed from the partial moments in Eqs. 29-57. Plugging in these expressions, we obtain the steady state mean and central moments 2-4. The mean and variance are printed here:

$$\langle q \rangle = \frac{t_{on}}{t_{on} + t_{off}} \frac{k_m k_p}{d_m d_p} \tag{S61}$$

$$\begin{aligned}
\mu_2(q) = &(k_p k_m t_{on} (d_p^2 (t_{off} + t_{on})(d_m + t_{off} + t_{on}) \\
&+ (d_m + t_{off} + t_{on}) \\
&(d_m (t_{off} + t_{on})^2 + k_p (k t_{off} + (t_{off} + t_{on})^2)) \\
&+ d_p (d_m^2 (t_{off} + t_{on}) + (t_{off} + t_{on})^3 + d_m (t_{off} + t_{on})(k_p + 2(t_{off} \\
&+ t_{on})) + k_p (k t_{off} + (t_{off} + t_{on})^2)))) / (d_p d_m (d_p + d_m)(t_{off} \\
&+ t_{on})^2 (d_p + t_{off} + t_{on})(d_m + t_{off} + t_{on}))
\end{aligned} \tag{S62}$$

Mean and variance Eqs. 65 and 62 agree with previously published work [2], though our solution is written completely in terms of the CDRCs. This makes it less interpretable but more readily computed. The higher two moments written in terms of the CDRCs are extremely long. They are provided along

with this supplement coded in both C++ and Matlab for convenience.

An interesting observation is that d_m and d_p are completely interchangeable with their sum and product. That is, if we set:

$$\theta_d = d_m + d_p \tag{S63}$$

$$\lambda_d = d_m d_p \tag{S64}$$

we can completely eliminate the variables d_m and d_p from Eqs. 65 and 62.

$$\langle q \rangle = \frac{t_{on}}{t_{on} + t_{off}} \frac{k_m k_p}{\lambda_d} \tag{S65}$$

$$\begin{aligned} \mu_2(q) =& (k_p k_m t_{on} (\theta_d (t_{off} + t_{on})(\lambda_d + (t_{off} + t_{on})(\theta_d + t_{off} + t_{on})) + \\ & k_p(\lambda_d (t_{off} + t_{on}) + (\theta_d + t_{off} + t_{on})(k_m t_{off} + (t_{off} + t_{on})^2)))) \\ & /(\lambda_d \theta_d (t_{off} + t_{on})^2 (\lambda_d + (t_{off} + t_{on})(\theta_d + t_{off} + t_{on}))) \end{aligned} \tag{S66}$$

This result demonstrates that d_m and d_p do not independently contribute to the mean and variance of a gene's protein expression distribution, but rather their individual effects are convolved as a sum and a product. As such, there will always be at least two solutions given mean and variance only, unless d_m=d_p. We speculate this is the reason why many previous methods require the degradation rate assumption that d_m>d_p, as this allows one to select between two candidate solution pairs.

Notably, the parameter conversion described does not work for skewness or kurtosis. This indicates that skewness and kurtosis introduce an asymmetry that allows their individual effects to be recognized.

S2 Validation of moment equations

Moment equation implementations provided in this supplement have been tested against Gillespie simulations to confirm their accuracy. Example convergences of a Gillespie simulation to the analytical moment solutions are given in Fig. S1. In this figure, relative error is computed as $|\mu_j - c_j(t)|/\mu_j$ where $c_j(t)$ is the j-th sample moment of the protein probability mass function computed at time t. Note that even

though the average protein count is low ($\langle q \rangle = 216$ proteins), the Gillespie simulation is extremely slow to converge; convergence occurs around 2 x 10^6 seconds, or 23.1 days (simulation time, not actual). Even after convergence, the probability mass function exhbits small but significant excursions away from the expected value.

Like in Fig. S1, we find Gillespie converges on our analytical solution regardless of what parameters are used. We conclude that the analytical solutions for all moments are correct.

S3 Analytically Constrained Exhaustive Search (ACES)

To derive CDRCs from the moments we wrote a numerical search algorithm. Applying a general-purpose fitting algorithm like traditional least-squares steepest descent, or even stochastic approaches like genetic or simulated annealing algorithms proved problematic. First, optimum behavior of most general purpose fitters require an accurate gradient. However, fitting on multiple moments with unknown derivatives makes gradient calculations challenging. It is also unclear how to weight each of the moments in the objective function; for example, is it better to have the mean moment off by 2% and the skewness off by 20%, or the mean off by 3% and the skewness 10%? Moreover, we expected our fitting to be underdetermined in most, if not all scenarios.

The ACES algorithm performs an exhaustive search of CDRC parameter space between each parameter's minimum and maximum value, where each CDRC range has been estimated from the literature (see main document). Given these boundary values for each CDRC, we subdivide the range equally in log space according to a resolution R and test every combination of each parameter value with every other parameter value. Effectively we test R^6 CDRC combinations in an ACES run at resolution R. For clarity we discuss below the first step of the ACES algorithm in the Materials and Methods section. The full pseudocode and a more detailed explanation is then provided below. Source code for ACES is provided in a seprate file.

S3.1 Algorithm

Procedure ACES(Mean$_{input}$, Variance$_{input}$, Skewness$_{input}$,Kurtosis$_{input}$)

M←Mean$_{input}$, V←Variance$_{input}$, S←Skewness$_{input}$, K←Kurtosis$_{input}$

Ranges [d_{p-min}, d_{p-max}], [d_{m-min}, d_{m-max}], [k_{p-min}, k_{p-max}],

[k_{m-min}, k_{m-max}], [t_{on-min}, t_{on-max}], [$t_{off-min}$, $t_{off-max}$] (physiological ranges)

R$\leftarrow$resolution

tol$\leftarrow$relative error permitted in moment

S$\leftarrow\{\}$ (solution list)

Vkm$\leftarrow$N_log_space(k_{m-min},k_{m-max},R)

for all $i \in$ Vkm

$kp'\_min \leftarrow \mathtt{M}\frac{d_{p-min}d_{m-min}(t_{on-max}+t_{off-min})}{\mathtt{Vkm(i)}t_{on-max}}$

$kp'\_max \leftarrow \mathtt{M}\frac{d_{p-max}d_{m-max}(t_{on-min}+t_{off-max})}{\mathtt{Vkm(i)}t_{on-min}}$

$kp'\_min \leftarrow$ max(kp'_min,k_{p-min}), $kp'\_max \leftarrow$ min(kp'_max,k_{p-max})

Rreduce $\leftarrow \frac{kp'\_max-kp'\_min}{k_{p-max}-k_{p-min}}$

Vkp$\leftarrow$N_log_space(kp'_min,kp'_max,ceiling(R*Rreduce))

for all $j \in$ Vkp

$dm'\_min \leftarrow \frac{\mathtt{Vkm(i)Vkp(j)}t_{on-min}}{(t_{on-min}+t_{off-max})d_{p-max}}\frac{1}{\mathtt{M}}$

$dm'\_max \leftarrow \frac{\mathtt{Vkm(i)Vkp(j)}t_{on-max}}{(t_{on-max}+t_{off-min})d_{p-min}}\frac{1}{\mathtt{M}}$

$dm'\_min \leftarrow$ max(dm'_min,d_{m-min}), $dm'\_max \leftarrow$ min(dm'_max,d_{m-max})

Rreduce $\leftarrow \frac{dm'\_max-dm'\_min}{d_{m-max}-d_{m-min}}$

Vdm$\leftarrow$N_log_space(dm'_min,dm'_max,ceiling(R*Rreduce))

for all $x \in$ Vdm

$dp'\_min \leftarrow \frac{\mathtt{Vkm(i)Vkp(j)}t_{on-min}}{(t_{on-min}+t_{off-max})\mathtt{Vdm(x)}}\frac{1}{\mathtt{M}}$

$dp'\_max \leftarrow \frac{\mathtt{Vkm(i)Vkp(j)}t_{on-max}}{(t_{on-max}+t_{off-min})\mathtt{Vdm(x)}}\frac{1}{\mathtt{M}}$

$dp'\_min \leftarrow$ max(dp'_min,d_{p-min}), $dp'\_max \leftarrow$ min(dp'_max,d_{p-max})

Rreduce $\leftarrow \frac{dp'\_max-dp'\_min}{d_{p-max}-d_{p-min}}$

Vdp$\leftarrow$N_log_space(dp'_min,dp'_max,ceiling(R*Rreduce))

for all $y \in$ Vdp

pstar $\leftarrow \mathtt{M}\frac{\mathtt{Vdm(x)\ Vdp(y)}}{\mathtt{Vkm(i)\ Vkp(j)}}$

$ton'\_min \leftarrow \frac{\mathtt{pstar}\ t_{off-min}}{1-\mathtt{pstar}}$

$ton'\_max \leftarrow \frac{\mathtt{pstar}\ t_{off-max}}{1-\mathtt{pstar}}$

$ton'\_min \leftarrow$ max(ton'_min,t_{on-min}), $ton'\_max \leftarrow$ min(ton'_max,t_{on-max})

Rreduce $\leftarrow \frac{ton'\_max-ton'\_min}{t_{on-max}-t_{on-min}}$

```
Vton←N_log_space(dp'_min,dp'_max,ceiling(R*Rreduce))
for all z ∈ Vton
    t_off ← (pstar Vton(z))/(1−pstar)
    candidate_cdrc ← [Vton(z) t_off Vkm(i) Vkp(j) Vdm(x) Vdp(y)]
    if |V−Varsoln(candidate_cdrc)|/V <tol and |S−Skewsoln(candidate_cdrc)|/S <tol
        and |K−Kurtsoln(candidate_cdrc)|/K <tol
        S ← cand_cdrc
return S
```

function N_log_space(MIN, MAX, number_subdivisions)

```
% Computes number_subdivisions values evenly spaced in log space from MIN to MAX,
% returns a vector containing these values
    stepSize=(log(MAX)-log(MIN))/(number_subdivisions-1)
    out_vector←{}
    for i=0 to number_subdivisions-1
        out_vector[i]=10^(log(MIN)+stepSize*i)
    return out_vector
```

In short, ACES sub-divides the first parameter k_m into R values from k_{m-min} to k_{m-max} spaced evenly in log space (`function N_log_space()`). We called this vector `Vkm`. We then iterate through that vector. For each value of `Vkm(i)`, we compute the ranges of possible values for the next parameter, k_p. By default, k_p will range from its min to its max, as defined by the physiological ranges (user input). However, given that the particular k_m value is fixed at `Vkm(i)`, and that the mean is fixed, it is now possible that certain values within the full physiological range of k_p cannot satisfy the mean equation. The equations immediately following each for loop compute the maximum and minimum values of the current parameter *given the parameters that are fixed at that point in the algorithm* (i.e., all preceding parameters).

When t_{off} is reached, we have one equation and one unknown; the remaining parameters are fixed. Upon solving for t_{off}, we have a candidate CDRC solution set that is guaranteed to satisify Eq. 65

where the mean=M_{input}. We then check higher moments. The functions `Varsoln()`, `Skewsoln()`, and `Kurtsoln()` take a set of CDRCs as inputs and return the moment solution.

In principle, the order in which the parameters are checked should not matter. However, we found that by random chance the order will matter since order changes exactly which values are placed in the CDRC test sets. This difference becomes less substantial as the resolution R increases, as expected. For the resolutions tested (usually R=125), order did not affect the MD values obtained.

ACES differs in behavior from a conventional fitting algorithm. ACES is deterministic; that is given the same input moments, physiological ranges for CDRCs, resolution and moment tolerance, ACES will always return the same set of results. This is similar to a steepest-descent fitting algorithm like traditional least-squares, but differs in that all fits, rather than one fit, are returned. In principle if we ran least-squares many times, with initial guesses spanning the same CDRC test sets as in ACES, we might obtain a similar result. However, least squares operates on the assumption that the solution that best minimizes the objective function is the best fit. We found that among the many solutions returned by ACES, all of which satisfy the moment tolerance cutoff, proximity in moment space does not predict proximity in parameter space. That is, among all solutions that satisfy our relative error of moment <1% threshold, the solutions whose moments closest match the input moments fail to predict the solutions closest in CDRC space. In principle, as $R \to \infty$ we should eventually test the true solution set which will exactly match the input moments; but at the resolutions we tested, any solution satisfying our threshold was equally likely to be closest to the true CDRC set.

S3.2 Reverse order algorithm

Fitting ACES on our library revealed a subset of input CDRC sets for which we could not find any solution. While the low initial resolution of 83 left about 500 library members without a solution, increasing the resolution marginally to 127 proved to be enough to fit the majority of the 500. However, 48 remained without solutions, and even raising resolution as high as 773 yielded solutions for only a few.

These input sets share several features; all had a negative third moment, and all pairs of t_{on} and t_{off} yielded a $P_{on} \approx 1$. In the original ACES formulation, the order of CDRC scanning was k_m, k_p, d_m, d_p, then t_{on} and t_{off}. We found that even when ACES proposed CDRC sets where the first four CDRCs in the algorithm were almost exact, t_{on} and t_{off} were always off by several orders of magnitude, resulting in candidate moment sets where skewness was positive rather than negative. This problem arises because

when $t_{on} \gg t_{off}$, exactly how much t_{on} is greater than t_{off} has a miniscule impact on P_{on}, but a large impact on distribution skewness. Thus, small errors in the guesses for the first four parameters propagate into massive errors in t_{on} and t_{off}.

To address this, we generated a second version of ACES where we reversed the order in which parameters are tested. With t_{on} and t_{off} tested first, their search is unbiased by choices of the other four parameters. This allowed ACES to find solutions for all remaining input sets at low resolutions (83 or 127).

The reverse-order ACES formulation appears to be about three times slower than the original formulation. We also did not rerun our the remaining library using this version of ACES. For these reasons, we suggest the original formulation is used at increasing resolutions up to two hundred or three hundred subdivisions. Above that, we recommend testing whether the reverse order formulation recovers solutions at lower resolutions.

S4 CDRC input library

We have an analytical solution for producing moments from CDRCs, and an inverse solver called ACES that attempts to find candidate CDRC sets consistent with a set of moments. To test ACES we generated a library of test CDRC sets. For each CDRC set we calculate their theoretical moments using the analytical solutions. Then, fitting only on these resulting moments, we ran ACES.

Since ACES should work regardless of parameter regime, we generated a course grained library of CDRC input sets where every rate constant is varied across its entire physiological range, and we test every combination of these subject to the min and max protein counts discussed in the paper. Since ACES follows a similar protocol–that is, ACES tests every combination of each parameter across its physiological range–we were careful not to generate input sets that were guaranteed to be exactly tested by ACES. For example, if the physiological max value for k_m were .01, and this was also one of the course grained input values for k_m, ACES is guaranteed to test exactly $k_m = .01$ and would give falsely high estimations of CDRCs from moments. To ameliorate this we generated evenly distributed values in log space of each parameter across their physiological range scaled from each ranges $min * 1.5$ and $max/1.5$.

The library of CDRC input sets is then every combination of values for a row with all other values in other rows. For example, input set 1 might be value 1 for all parameters. Input set 2 might be value

Table S1. CDRC Library Input Values

	value 1	value 2	value 3	value 4	value 5
t_{on}	0.0015	0.0183	0.2236	2.7301	33.3333
t_{off}	0.0015	0.0183	0.2236	2.7301	33.3333
k_m	0.015	0.1456	1.4142	13.7318	133.3333
d_m	0.0009	0.0064	0.0441	0.302	2.0667
k_p	0.75	1.9832	5.244	13.8666	36.6667
d_p	0.0007	0.0038	0.0212	0.1189	0.6667

1 for all CDRCs except d_p, which takes on value 2. Given 6 CDRCs and 5 possible values for each, we get $5^6 = 15625$ possible CDRC sets, of which 8053 result in sets that have a mean protein count between 17 and 100,000. The supplemental file called CDRC_library.sh is a text file (or shell script) that lists the 8053 input sets. See README_aco_known.txt for a description of how to use this as a shell script to run the whole library through ACES.

S5 Contribution of the 5th moment

One of the central findings in this paper is that four moments appears to completely capture the shape of a distribution. However, there are subtle differences between these distributions. A reasonable question to ask is whether measuring a 5th moment could distinguish good CDRC sets from poor CDRC sets that have the same first four moments.

To address this question, we performed the following analysis on the Gillespie-simulated distributions of our "maximally distant" CDRC sets and their reference. First, we calculated the fifth sample non-normalized central moment for each distribution. Then we computed the relative error between the 5th moment of each maximally distant set distribution and its reference. We find that the average relative error of a maximally distant set with its reference was .073 $\pm$ .061. The high variance is consistent with some sets showing modest differences in their fifth moments (as high as .27), while the majority demonstrate that the fifth moments are identical. This is consistent with our observation that the distributions measured in this experiment are roughly identical.

One problem is that a large number of observations are required to accurately estimate higher order moments. We found we could reliably estimate kurtosis with the Gillespie algorithm when distributions were estimated from one million observations. However, we asked whether the differences in the 5th moment, which are already quite small, were significantly different from the differences we might observe

from two independent Gillespie simulations of the same CDRC set. We chose the reference CDRC for each of our maximally distant pairs and re-simulated each. We found that the relative error between the 5th moment of two distributions that were simulated *from the same CDRC set* was $.08 \pm .072$, essentially the same difference as between distributions generated from CDRC sets that have different rate constants but identical moments.

We therefore conclude that the fifth moment for these maximally distant pairs and their reference are indistinguishable, at least when simulating distributions with a million observations. It is possible the fifth moment could provide additional information if we derived finer resolution distributions by simulating more observations. However, measuring expression in even a million cells is approaching–or beyond–the upper limit of what is measurable experimentally by flow cytometry, and significantly beyond the number measurable by microscopy.

S6 Gillespie simulations

We implemented Gilllespie's direct algorithm [3] in C++ for all simulations. After verifying the accuracy of analytical solutions for the first four moments, we used agreement between these analytical solutions and distribution sample moments as convergence criteria. To ensure that convergence was stable, after each initial convergence we continued the simulation for a discrete period and then re-tested for agreement between sample and analytical moments.

S7 Identifying gamma distributions across the library

The most direct way of identifying which CDRC sets correspond to gamma distributions would be to Gillespie simulate all library members. We quickly realized this was intractable, as generating high enough quality probability mass functions by Gillespie simulation proved too time-intensive. For CDRC sets corresponding to average protein counts over 10000, simulation took on the order of an hour just to rule out a distribution being gamma-shaped, and much longer to confirm a gamma distribution.

To avoid Gillespie-simulating all 8053 library input CDRC sets, we leveraged the known analytical moments of a gamma distribution as a screening tool. Gamma distribution parameters k and θ can be computed directly from the mean and variance: $k = \mu_1^2/\mu_2$, $\theta = \mu_2/\mu_1$, where μ_i is the ith central

moment. For each library input set we can derive the corresponding candidate k^* and θ^*. If a CDRC set results in a gamma distribution, we expect agreement between gamma distribution skewness ($\mu_3/\mu_2^{\frac{3}{2}} = 2/\sqrt{k}$) and CDRC derived skewness from our analytical result. We further checked our work by (1) comparing candidates' gamma distribution excess kurtosis ($\mu_4/\mu_2^2 - 3 = 6/\sqrt{k}$) with our analytically derived excess kurtosis, and (2) Gillespie-simulating low protein count example CDRC sets to generate high quality probability mass functions. Both exercises revealed excellent agreement with the predicted gamma distribution.

Results of this analysis are reported in Data 1, sheet MVSK, in far right columns.

References

[1] Sánchez, A. & Kondev, J. Transcriptional control of noise in gene expression. *Proceedings of the National Academy of Sciences of the United States of America* **105**, 5081–6 (2008). URL `http://www.pubmedcentral.nih.gov/articlerender.fcgi?artid=2278180&tool=pmcentrez&rendertype=abstra`

[2] Paulsson, J. Models of stochastic gene expression. *Physics of Life Reviews* **2**, 157–175 (2005). URL `http://linkinghub.elsevier.com/retrieve/pii/S1571064505000138`.

[3] Gillespie, D. T. A general method for numerically simulating the stochastic time evolution of coupled chemical reactions. *Journal of Computational Physics* **22**, 403–434 (1976). URL `http://linkinghub.elsevier.com/retrieve/pii/0021999176900413`.

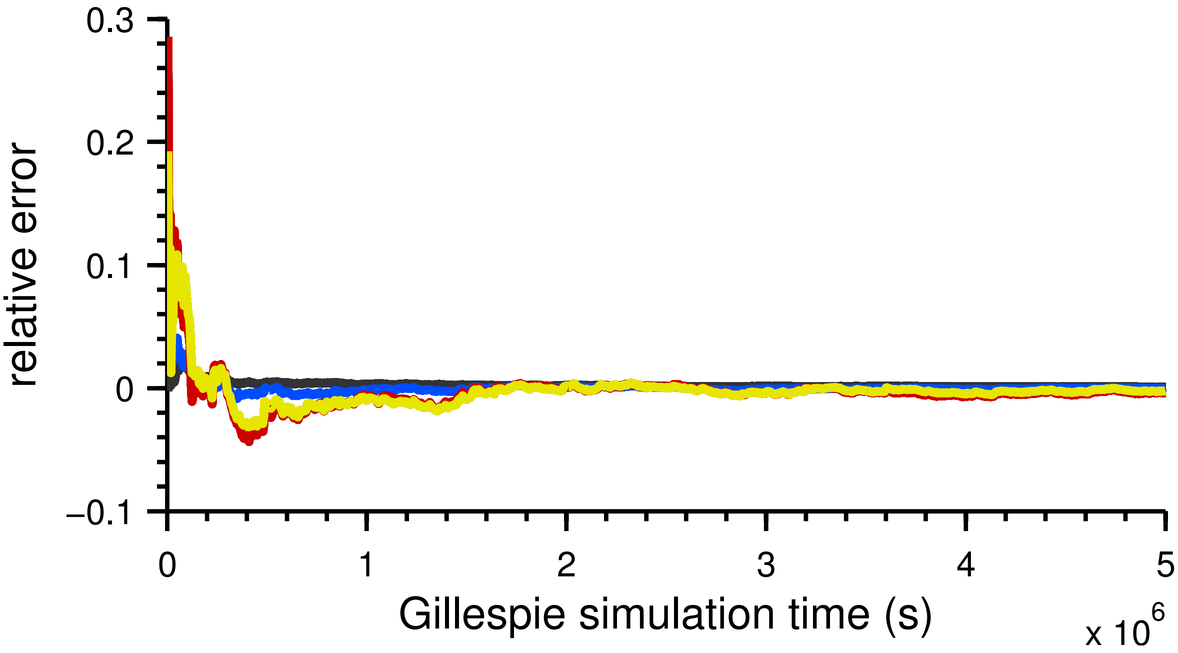

Figure S1. Convergence of sample moments on analytical moments. Example Gillespie simulation of parameter set with CDRCs: $t_{on} = 2.5s^{-1}$ $t_{off} = 10s^{-1}$ $k_m = 3s^{-1}$ $d_m = .5s^{-1}$ $k_p = 18s^{-1}$ $d_p = .1s^{-1}$. Plotted is the running relative error of the sample moment from the Gillespie simulation with respect to the analytical moment solution. The four traces correspond to Mean (black), Variance (blue), Skewness (red), Kurtosis (yellow).

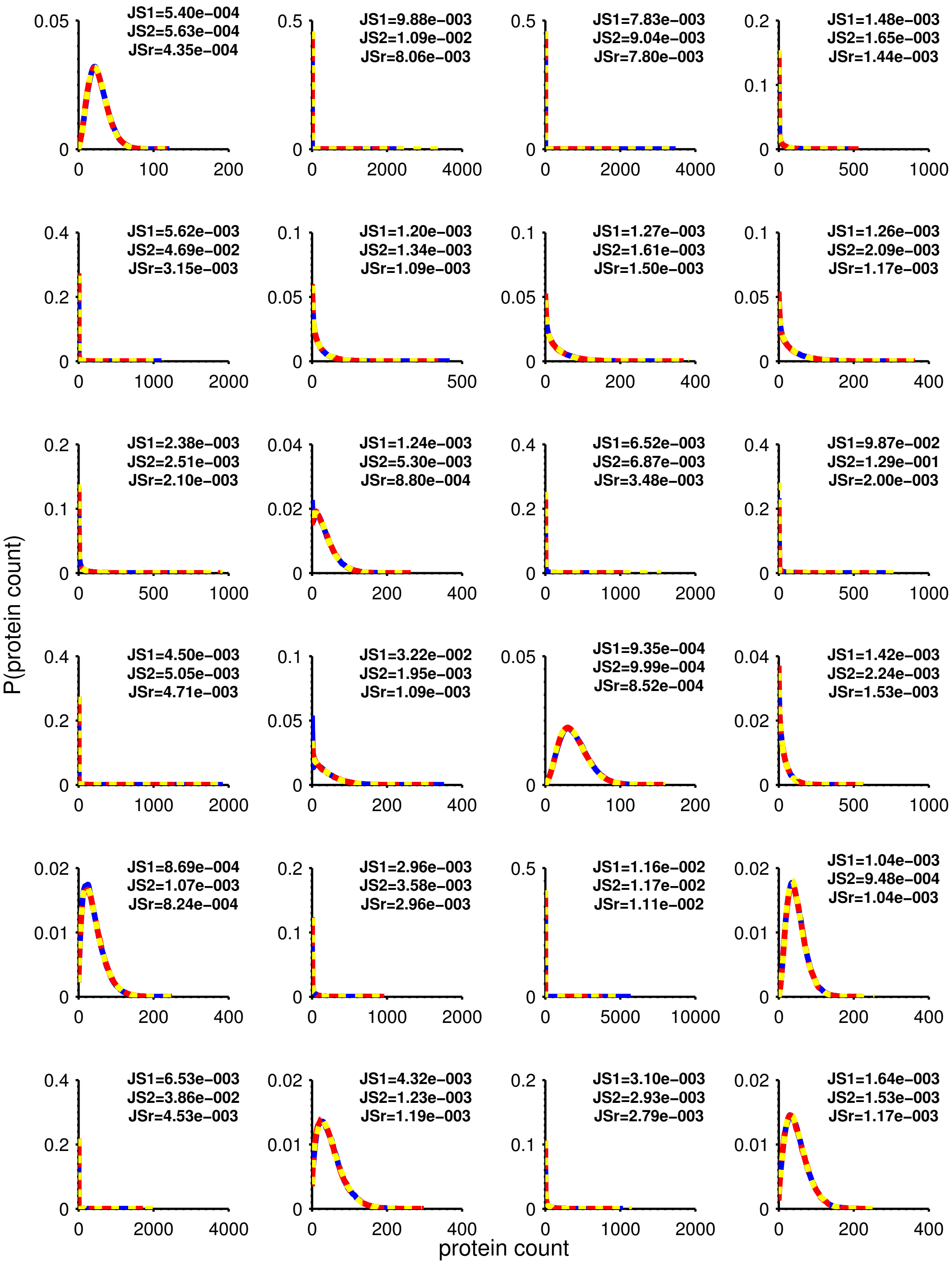

JS1=5.40e−004
JS2=5.63e−004
JSr=4.35e−004
JS1=9.88e−003
JS2=1.09e−002
JSr=8.06e−003
JS1=7.83e−003
JS2=9.04e−003
JSr=7.80e−003
JS1=1.48e−003
JS2=1.65e−003
JSr=1.44e−003
JS1=5.62e−003
JS2=4.69e−002
JSr=3.15e−003
JS1=1.20e−003
JS2=1.34e−003
JSr=1.09e−003
JS1=1.27e−003
JS2=1.61e−003
JSr=1.50e−003
JS1=1.26e−003
JS2=2.09e−003
JSr=1.17e−003
JS1=2.38e−003
JS2=2.51e−003
JSr=2.10e−003
JS1=1.24e−003
JS2=5.30e−003
JSr=8.80e−004
JS1=6.52e−003
JS2=6.87e−003
JSr=3.48e−003
JS1=9.87e−002
JS2=1.29e−001
JSr=2.00e−003
JS1=4.50e−003
JS2=5.05e−003
JSr=4.71e−003
JS1=3.22e−002
JS2=1.95e−003
JSr=1.09e−003
JS1=9.35e−004
JS2=9.99e−004
JSr=8.52e−004
JS1=1.42e−003
JS2=2.24e−003
JSr=1.53e−003
JS1=8.69e−004
JS2=1.07e−003
JSr=8.24e−004
JS1=2.96e−003
JS2=3.58e−003
JSr=2.96e−003
JS1=1.16e−002
JS2=1.17e−002
JSr=1.11e−002
JS1=1.04e−003
JS2=9.48e−004
JSr=1.04e−003
JS1=6.53e−003
JS2=3.86e−002
JSr=4.53e−003
JS1=4.32e−003
JS2=1.23e−003
JSr=1.19e−003
JS1=3.10e−003
JS2=2.93e−003
JSr=2.79e−003
JS1=1.64e−003
JS2=1.53e−003
JSr=1.17e−003
P(protein count)
protein count

Figure S2. to S6. Gillespie simulations of maximally distant CDRC sets with the same moments. (1-24) Each plot represents one of each of the 99 most poorly fit distributions, determined by ranking the highest average MD for the CDRCs. In each graph, the probability mass functions are plotted for the reference input CDRC set (yellow dashed), and the two maximally disimilar CDRC sets from the ACES fit (red dashed, blue solid). In the top right corner, the Jensen-Shannon divergence is listed for comparing the reference versus the red distribution (JS1), reference versus the blue distribution (JS2), and reference versus an independent Gillespie-simulated replicate of the reference (JSr). The replicate reference distribution is not shown.

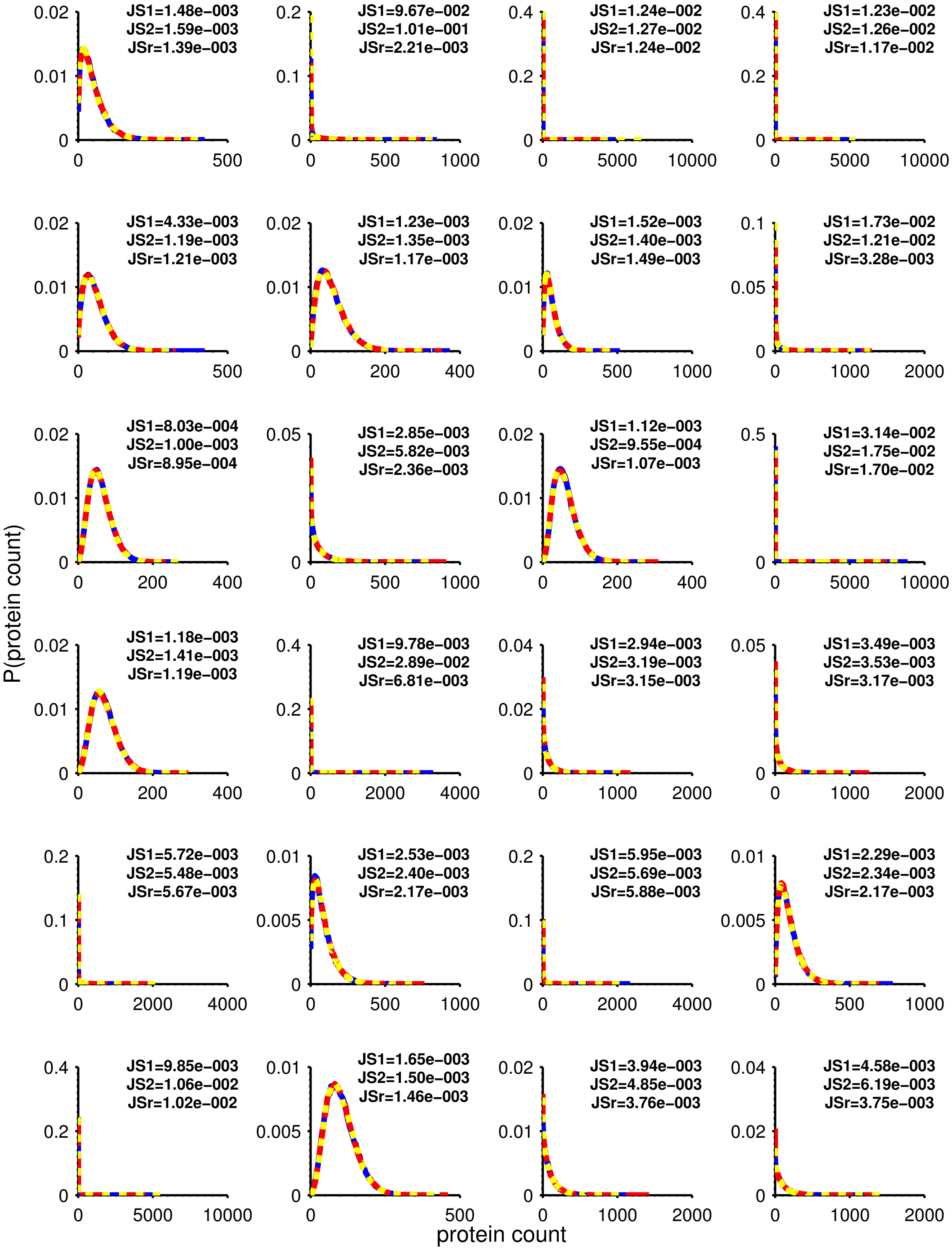

Figure S3. Gillespie simulations of maximally distant CDRC sets. (25-48)

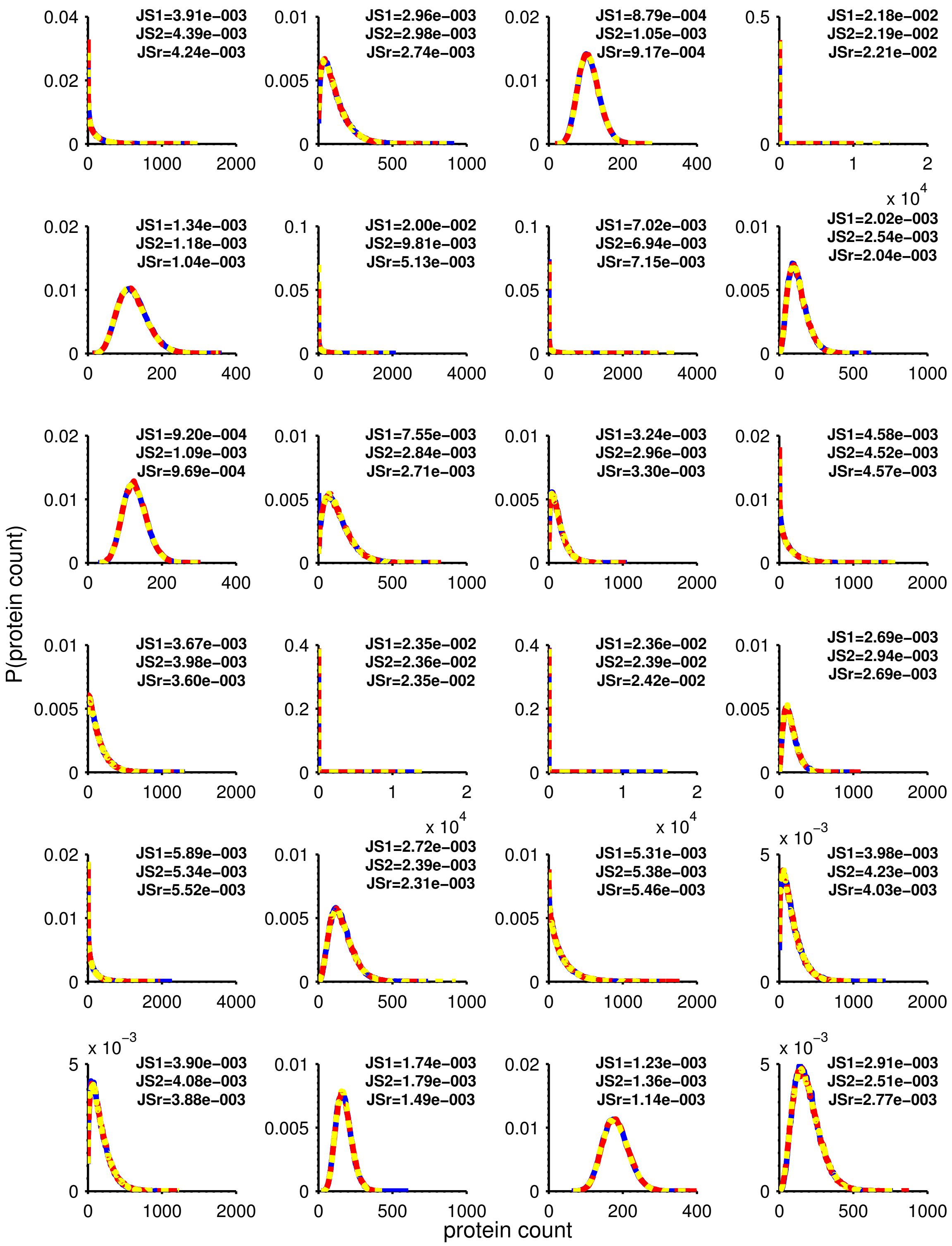

Figure S4. Gillespie simulations of maximally distant CDRC sets. (49-72)

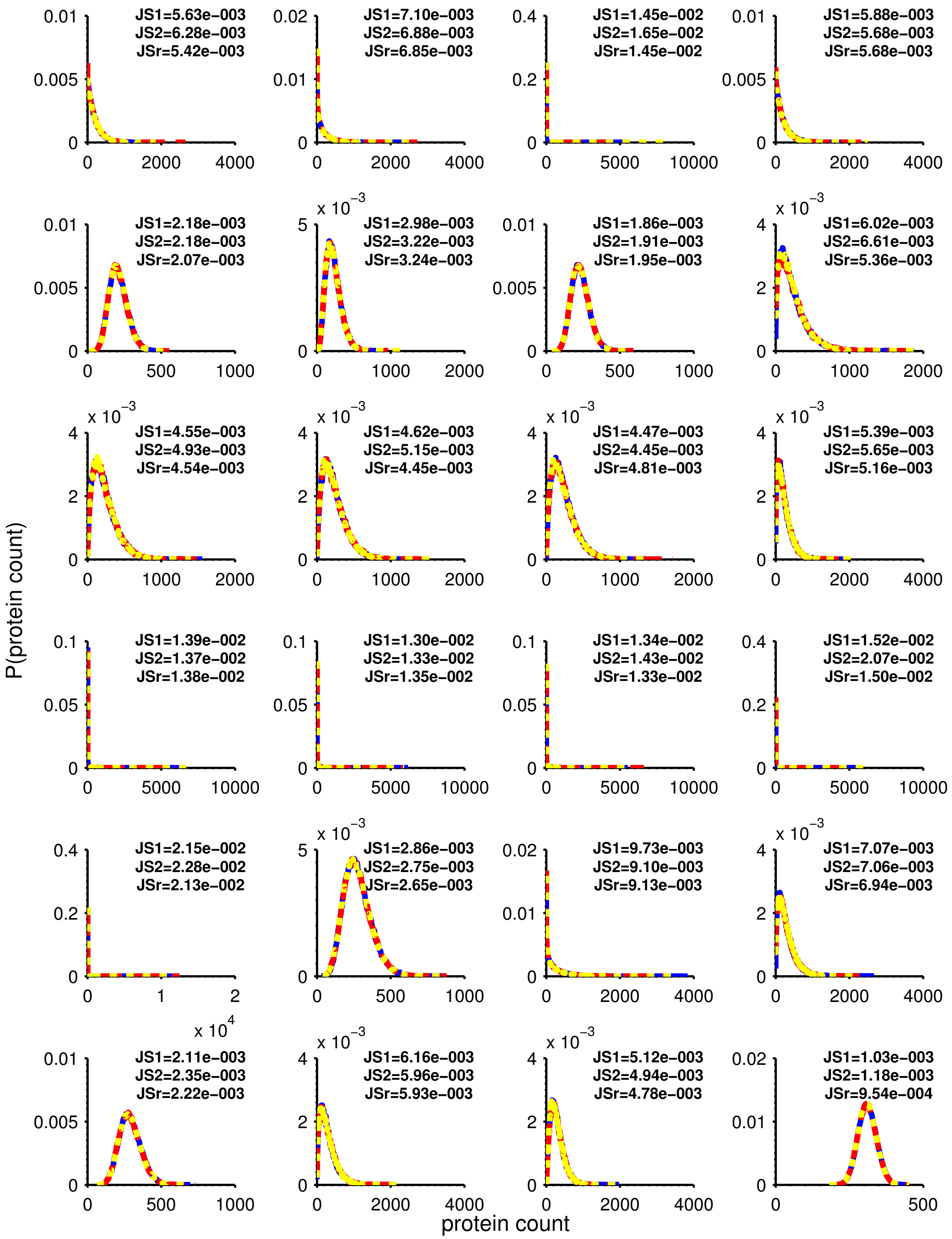

Figure S5. Gillespie simulations of maximally distant CDRC sets. (73-96)

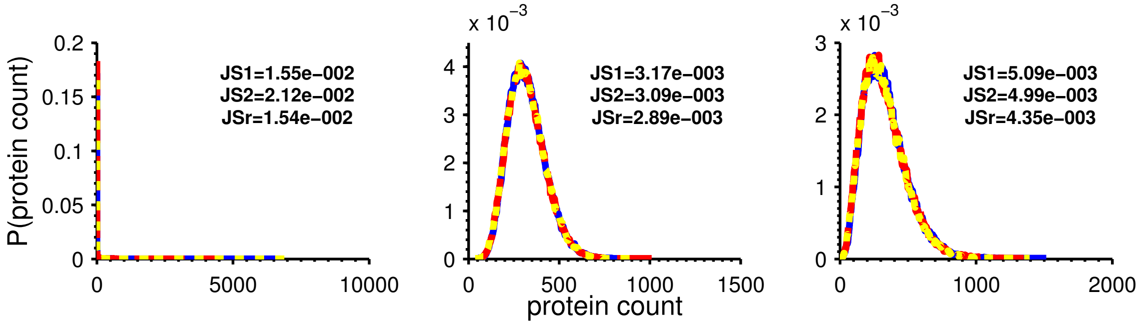

Figure S6. Gillespie simulations of maximally distant CDRC sets. (97-99)

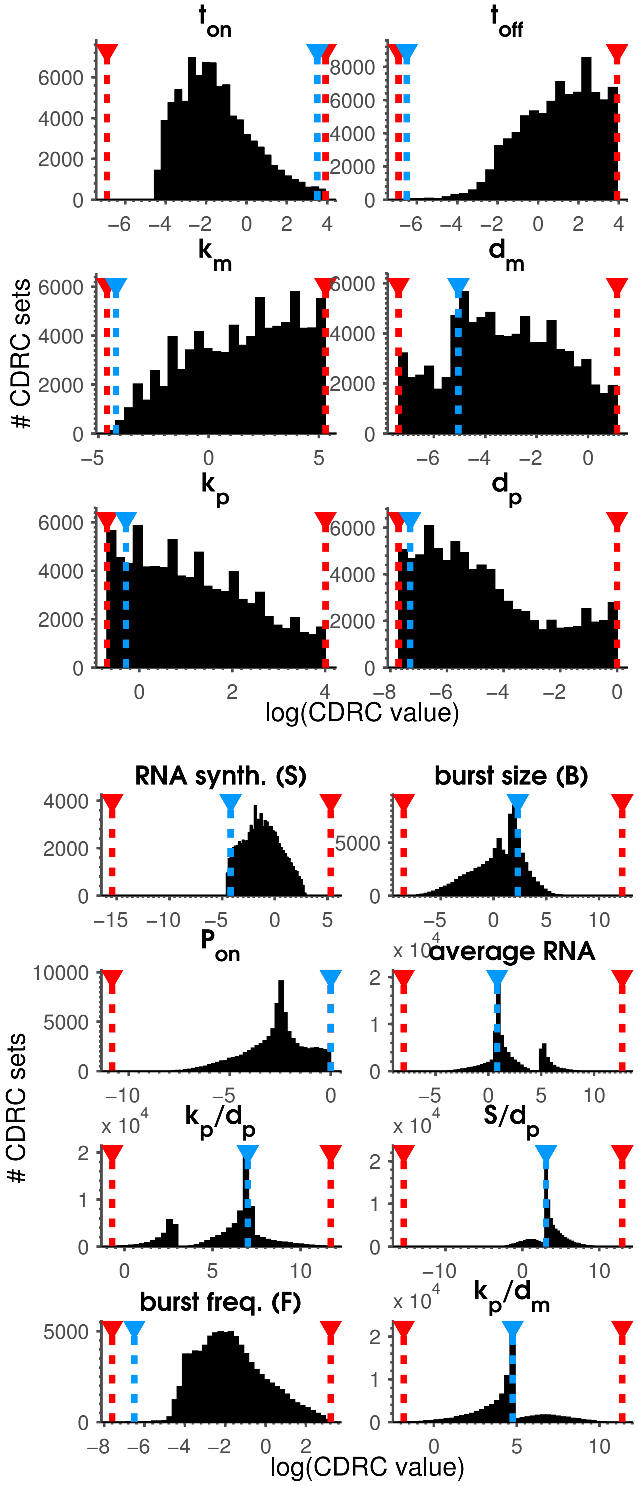

Figure S7. Library member 6353 has the highest average CDRC MD. CDRC output solution histograms are plotted in the top three rows, while their ratios are plotted in the bottom four rows.

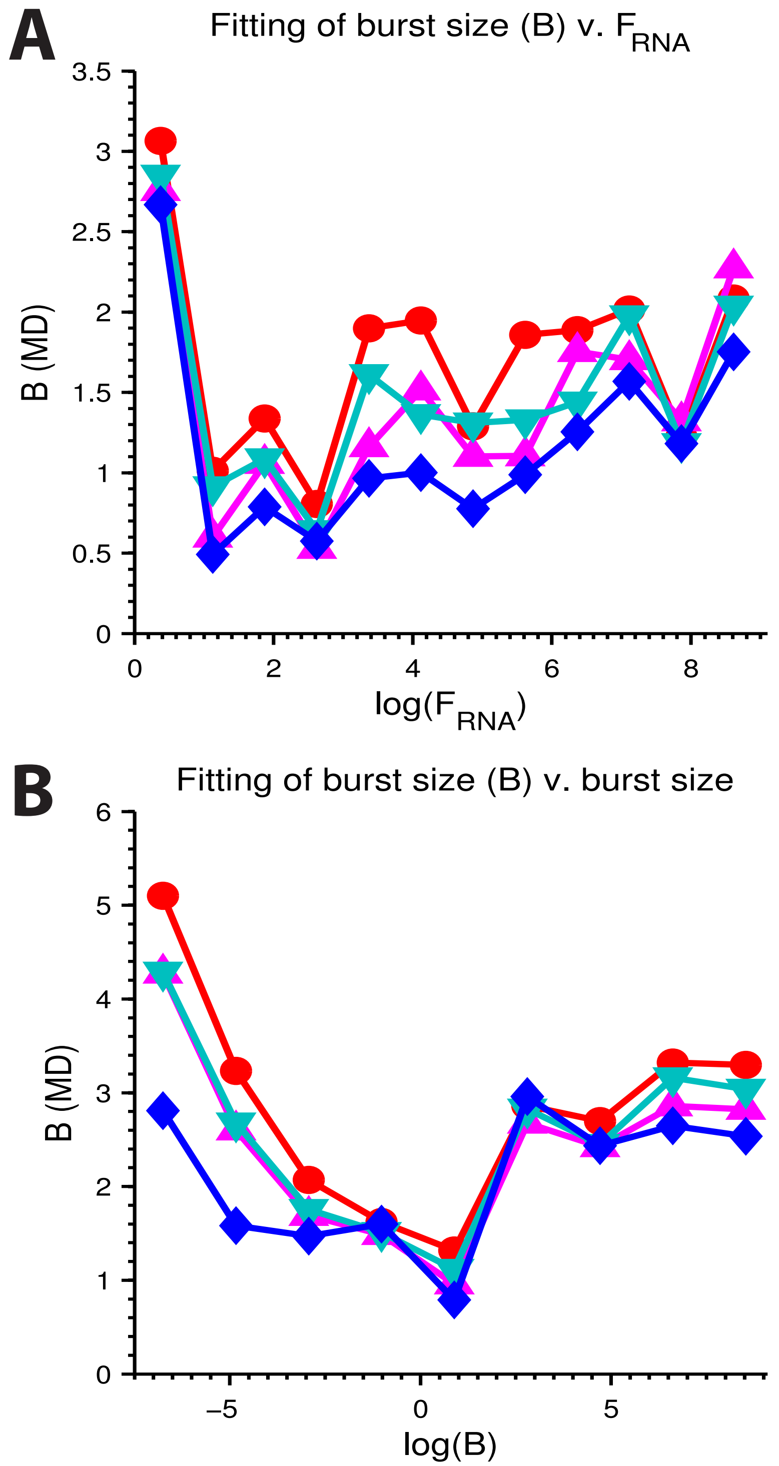

Figure S8. Fitting the burst parameter across regimes. Both plots show trends for the library where all four moments are known (red), where d_m (magenta) or d_p (cyan) is also known, and when both degradation parameters are known (blue). Each y-value represents how well the burst parameter was fit in units of MD for a bin of x-axis values. (A) Log of the RNA Fano factor plotted against the MD for the burst parameter. (B) Log of the burst parameter's input value plotted against the MD fit of the same parameter.

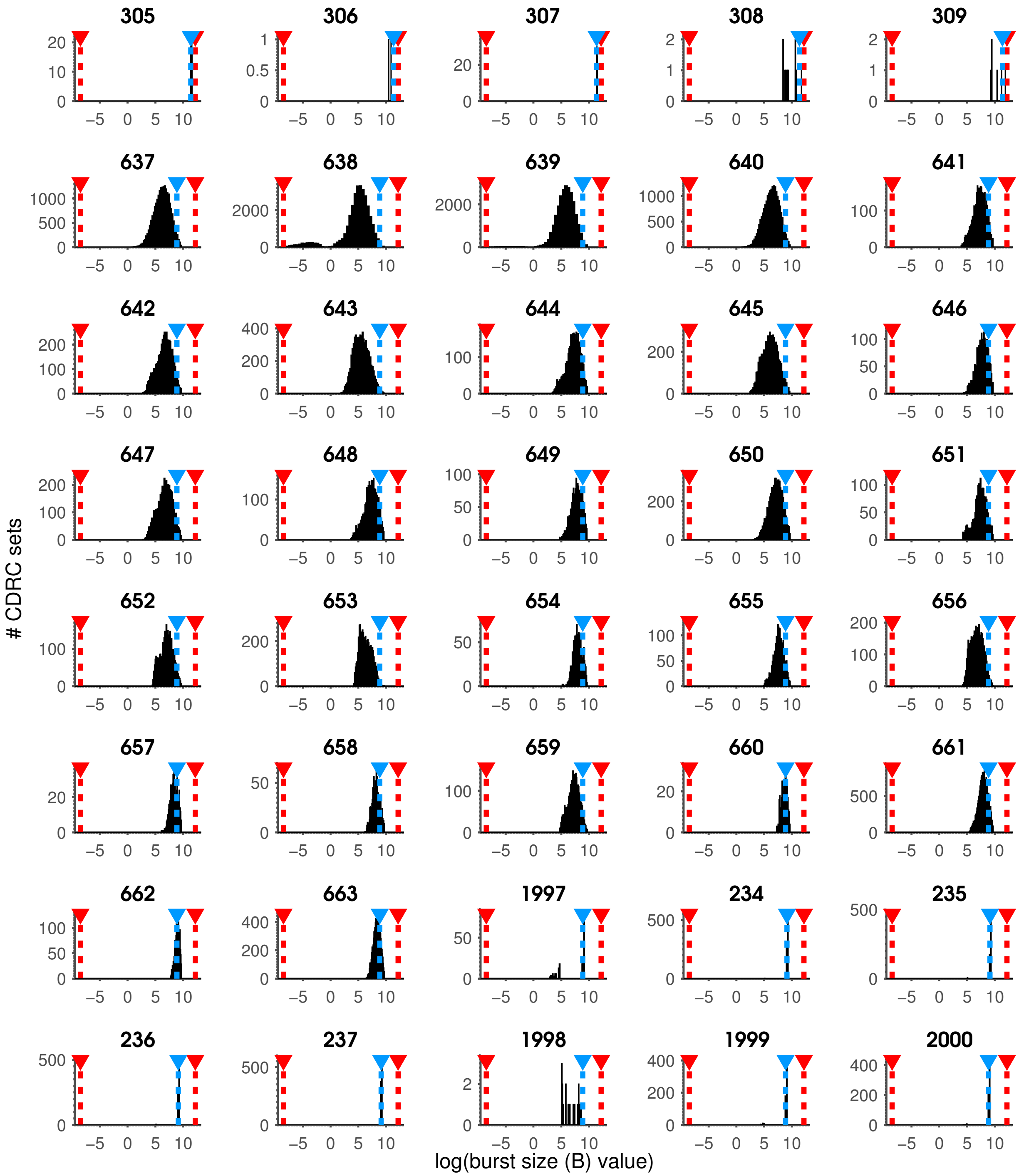

Figure S9. Solution ensembles for the burst parameter for high Fano factor library members. Plotted are the solution histograms for the burst size parameter for the library members corresponding to the top forty highest Fano factor RNA distributions, arranged in reading order from highest to lowest. Blue corresponds to the input value, red lines denote the range of the parameter.

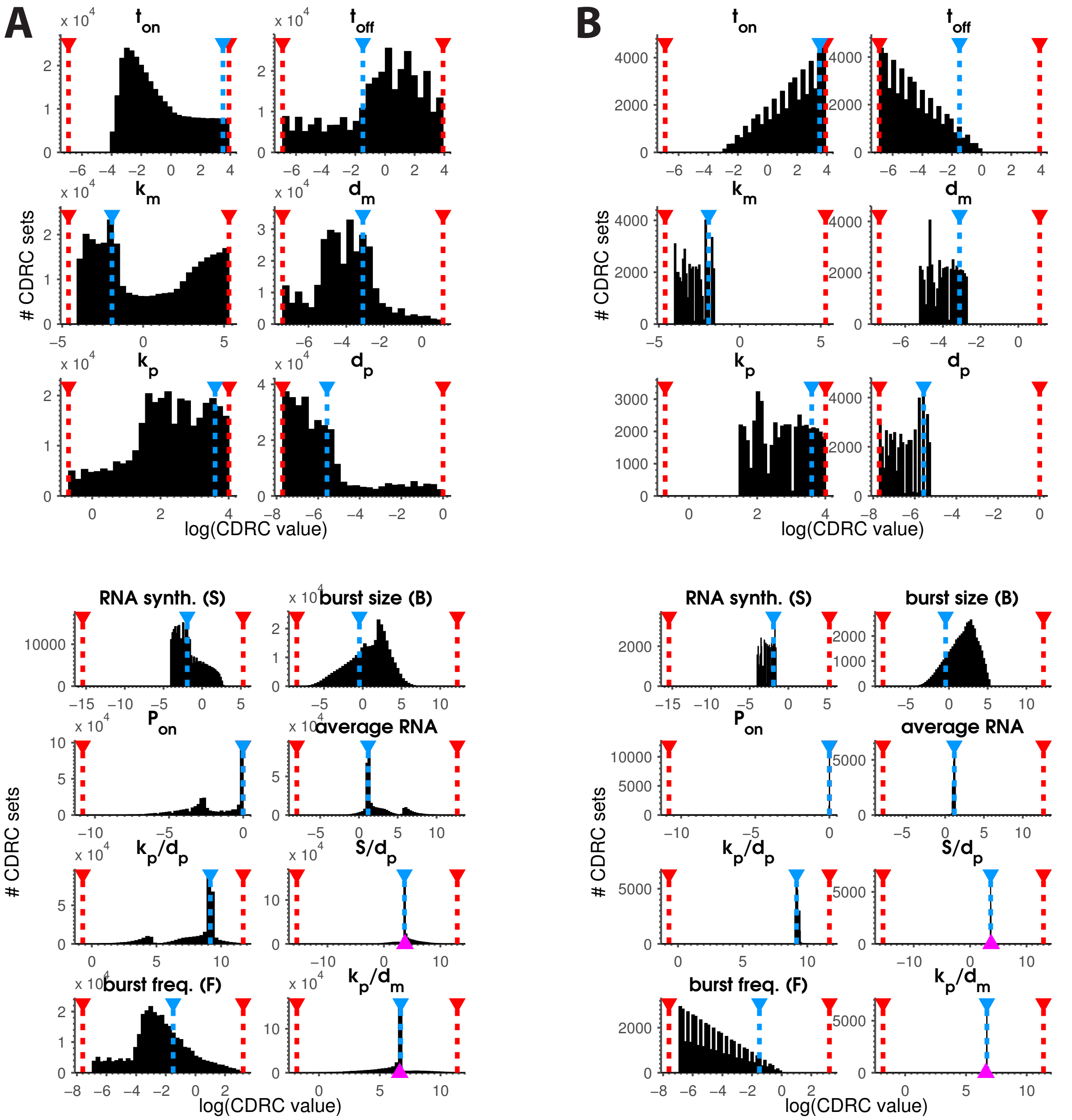

Figure S10. CDRC and ratio ensembles before and after assuming the Friedman regime. Plotted are the solution histograms for all CDRCs (top row) and ratios (bottom row) for library input set number 7144 before (A) and after (B) imposing that a given solution satisfies the Friedman assumptions: $d_m/d_p > 10$, average protein count >1000, and $t_{on} \gg t_{off}$ ($P_{on} \approx 1$). The Friedman ratios are $S/d_p = k_m/d_p$, and k_p/d_m. Analytical solutions from the Friedman result are marked with a magenta triangle.

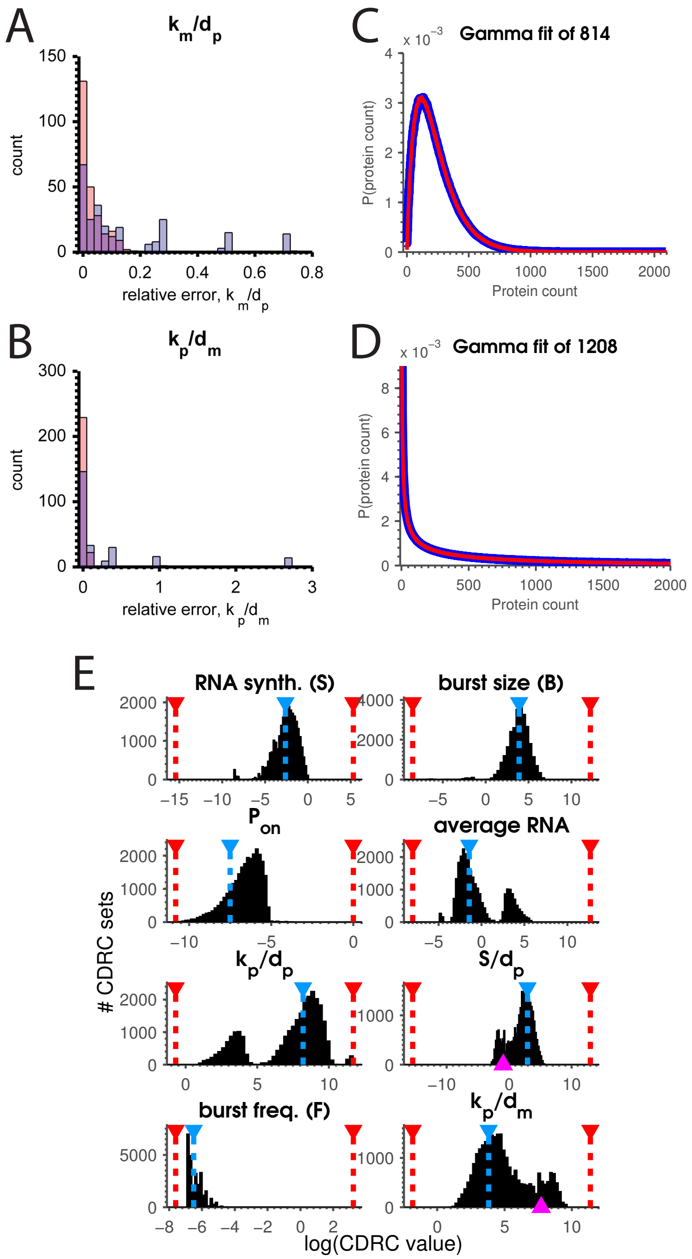

Figure S11. Fitting k_m/d_p and k_p/d_m by ACES versus the Friedman analytical solution. (A) Relative error in estimating k_m/d_p by ACES (red) versus the Friedman estimator when in the Friedman regime. Purple denotes overlap. (B) Relative error in estimating k_p/d_m by ACES (red) versus the Friedman estimator when in the Friedman regime. (C) Gillespie simulated probability mass function of input set number 814 (blue), which is both gamma distributed (red) and does not fall in the Friedman regime. (D) Gillespie simulated probability mass function of input set number 1208 (blue), which is both gamma distributed (red) and does not fall in the Friedman regime. (E) CDRC ratio histograms for input set 1208. The magenta triangles mark the predicted Friedman ratios.
